# Acoustic-Based Queen Bee Status Recognition: A Transfer Learning Approach Refinement

**DOI:** 10.3390/insects17060612

**Published:** 2026-06-10

**Authors:** Zidong Dai, Yurong Liu, Xiaoping Jiang

**Affiliations:** 1 School of Mechanical Electronic & Information Engineering, China University of Mining and Technology-Beijing, Beijing 100083, China; 18979680633@163.com; 2 School of Artificial Intelligence, China University of Mining and Technology-Beijing, Beijing 100083, China; liuyr_1024@163.com

**Keywords:** beehive monitoring, transfer learning, audio processing, generalization ability, ablation study, constant q transform, Grad-CAM

## Abstract

Honeybees are indispensable pollinators for agricultural ecosystems, and a colony’s stability and reproductive capacity depend critically on the presence of a healthy queen. Acoustic monitoring has emerged as a promising non-invasive, lighting-independent approach for long-term colony observation. However, existing studies have largely been confined to single-apiary datasets or merged datasets from multiple similar apiaries for model training. Moreover, model evaluation has relied primarily on overall performance metrics, with insufficient attention to cross-region generalization and the detection of queen loss, a rare but critical condition. This study systematically investigates three complementary strategies: noise-augmented data diversification, lightweight convolutional neural network (CNN) architecture optimization via comprehensive ablation experiments, and transfer learning with fine-tuning to bridge the domain gap between source and target apiaries. Under cross-apiary evaluation, the proposed approach achieves an accuracy of 92.79%, a negative-class F1-score of 0.7900, and a negative-class recall of 0.7834 when only limited target-domain training samples are available. With full target-domain training data, the same strategy further attains an accuracy of 95.05%, a negative-class F1-score of 0.8596, and a negative-class recall of 0.8733. t-distributed Stochastic Neighbor Embedding (t-SNE) visualization demonstrates that noise augmentation effectively expands sample diversity in the feature space, while Gradient-weighted Class Activation Mapping (Grad-CAM) heatmaps confirm the successful transfer of source-domain acoustic features to the target domain. This work provides a practical approach for deploying acoustic-based queen status monitoring across diverse apiaries with minimal local data collection.

## Simple Summary

Honeybees are key pollinators that ensure crop pollination and maintain ecological balance. The queen bee plays a critical role in maintaining colony health. Traditional manual hive inspections are labor-intensive and disturb the colony, whereas sound-based monitoring offers a non-invasive, automated alternative for long-term observation. However, existing sound recognition models are easily affected by environmental noise when deployed across different apiaries, and they often fail to accurately detect queen loss—a rare but serious condition. This study aims to develop a robust acoustic model for queen status recognition, with a particular focus on improving the detection of queen loss. The model achieved high accuracy under cross-apiary conditions and could promptly identify queen loss. Moreover, it can be deployed using only a small set of locally recorded sound samples, providing a feasible approach for cross-regional acoustic queen monitoring. This method can be integrated into smart apiary management systems, enabling beekeepers to remotely track queen status in real time, take timely action, and reduce colony losses, thus offering practical value for sustainable beekeeping and agricultural ecological security.

## 1. Introduction

Honeybees are regarded as one of the most important pollinating insects in agricultural ecosystems, serving an irreplaceable role in maintaining ecological balance and ensuring food production. According to available statistics, approximately 75% of the world’s major food crops depend on pollination by honeybees. Consequently, the health status of bee colonies is critically linked to agricultural security [1,2]. However, in recent years, the frequent occurrence of Colony Collapse Disorder (CCD), compounded by the combined pressures of climate change and pesticide use, has led to a continued decline in global honeybee populations, posing a real threat to ecological and agricultural systems [3,4,5,6]. Against this backdrop, how to achieve efficient and non-destructive monitoring of colony health status has become an urgent issue that needs to be addressed in apiculture and agricultural research [2].

In the study of colony health monitoring, queen bee status recognition plays a central role. The queen maintains the social structure of the colony by releasing pheromones and continuously laying eggs. Therefore, if the loss of a queen is not identified in time, the entire colony may collapse [7]. Traditional manual inspection through hive opening is inefficient and disrupts normal colony activities, hindering large-scale management. Given this limitation, non-invasive detection methods based on acoustic signals—which are not affected by lighting conditions or visual occlusion and can be deployed continuously over long periods—have emerged as a research focus.

The feasibility of using acoustic methods for colony monitoring was first indicated in the early work of Wenner [8] on sound communication in honeybees. Since then, studies by Cejrowski et al. [7] and Nolasco et al. [9] have further demonstrated that sound analysis can be used to identify the presence or absence of a queen. Quantifiable differences in acoustic features have been observed between queen-present and queen-loss colonies, and a complex communication system consisting of specific frequency and temporal patterns has been shown to exist within honeybee colonies [10,11,12,13,14]. These findings collectively provide the scientific foundation for non-invasive assessment of queen bee status using acoustic features.

The performance of queen bee status recognition is directly determined by the choice of acoustic feature extraction and classification models. Regarding feature extraction, Kulyukin et al. [15] systematically compared the effectiveness of Mel-Frequency Cepstral Coefficients (MFCCs), mel spectrograms (Mel-Spec), constant-Q transform (CQT), and Tonnetz features in beehive audio classification. Among these, MFCCs have been widely adopted due to their established success in speech recognition [12,15]. In addition, the CQT—characterized by its high resolution on a logarithmic frequency scale—is considered particularly suitable for bioacoustic signal analysis, given the harmonic structures often present in such signals. Nolasco et al. [12] further noted that frequency information is critical for identifying the presence or absence of a queen, thereby providing theoretical support for the application of the CQT in this context.

In terms of classification models, traditional machine learning methods such as support vector machines (SVMs) and Random Forests (RFs) have been widely applied to beehive audio classification tasks [12,15]. Meanwhile, convolutional neural network (CNNs) were employed by Nolasco et al. [9,14] for queen bee status recognition, with superior performance being achieved.

Despite the promising performance achieved by the aforementioned methods on single datasets or specific beehives, the accuracy and balanced performance of these models in cross-apiary scenarios remain a critical challenge. As noted by Cejrowski et al. [7] and Nolasco et al. [9], model performance degrades significantly when evaluated across different datasets or unseen beehives, indicating limited transferability. Abdollahi et al. [16] further corroborates this generalization issue; the review also notes that for inherently class-imbalanced tasks for queen absence detection, overall accuracy alone cannot adequately evaluate model performance, and greater attention to minority-class metrics is needed.

To systematically enhance the generalization performance of recognition models in cross-apiary scenarios with a focus on recognition capability under limited-sample conditions, this study implements methodological designs from three perspectives: data augmentation, architecture optimization, and transfer adaptation, with visualization analysis for validation. At the data level, noise augmentation is introduced into the source-domain training set to increase sample diversity and reduce the model’s reliance on specific background noises; meanwhile, the selection of acoustic features is discussed and optimized. t-SNE is employed for dimensionality reduction and visualization to illustrate the distribution of acoustic features in honeybee colonies, demonstrating, from the perspective of feature space, the expansion of the sample space achieved through augmentation and the separability of samples under different queen status conditions. At the model level, convolutional neural networks are established as the base model through comparative experiments, and the network architecture is progressively optimized through multiple rounds of experiments. At the training strategy level, model selection is guided by target-domain performance metrics; class imbalance strategies and learning rate configurations are analyzed; and different transfer strategies are systematically compared. Grad-CAM is employed to visualize the decision regions of the fine-tuned model, providing intuitive evidence from the perspective of feature focusing to validate the effective transfer of source-domain pre-trained features to the target domain, thereby enhancing the interpretability of transfer learning.

## 2. Materials and Methods

### 2.1. Data Sources

To evaluate the recognition performance of the acoustic-based queen status recognition model in cross-apiary scenarios, two public beehive audio datasets were employed in this study. The first is the “BeeHive Audio Dataset with Queen and without Queen” [17] (hereinafter referred to as BAD), which was designed for queen presence detection based on audio signals and spectrograms and can be used for acoustic event classification and bioacoustic monitoring research. The dataset consists of 4000 10 s samples with a queen present and 2000 10 s samples without a queen, along with 2000 5 s samples of motorcycle noise included as interference data. Only two classes—“queen present” and “queen absent”—were labeled in the dataset.

The second dataset is the “Smart Bee Colony Monitor: Clips of Beehive Sounds” [18] (hereinafter referred to as SBCM). It was collected from European honeybee hives in California using a custom-built IoT device integrating an ESP32 Wi-Fi module (Espressif Systems, Shanghai, China), an INMP441 microphone (InvenSense, Inc., San Jose, CA, USA), and a BME280 temperature and humidity sensor (Bosch Sensortec, Reutlingen, Germany). This dataset contains approximately 7100 samples, with a total duration of 118 h, and all audio clips are stored as 60 s segments. According to the dataset authors, SBCM records the queen presence status as a binary indicator, where a value of 1 denotes the presence of a queen and 0 denotes queen absence. Its recordings span a longer time period and cover varying environmental conditions compared to BAD, resulting in more complex inter-sample variability. Consequently, SBCM presents a more challenging classification task, and together with BAD provides a suitable framework for investigating cross-apiary transfer learning: BAD serves as the source domain for model pre-training, while SBCM serves as the target domain for adaptation.

### 2.2. Audio Preprocessing and Data Augmentation

To unify the input format and improve the recognition accuracy and balanced performance of the model in cross-region scenarios, all audio recordings were uniformly resampled to 16 kHz and then either truncated or zero-padded to a fixed duration of 5 s to ensure consistent sample length after validation.

After truncation or zero-padding to a fixed duration of 5 s, the BAD dataset yields 6000 queen-status samples (4000 positive, 2000 negative), while the SBCM dataset yields approximately 66,000 5 s clips. The 2000 motorcycle noise recordings from BAD are reserved for data augmentation via noise superposition. All these samples are used for subsequent feature extraction and model evaluation.

For feature extraction, the audio signals were first divided into frames and windowed. A frame length of 2048 samples (128 ms) and a hop length of 512 samples (32 ms) were adopted, resulting in a 75% overlap between consecutive frames to maintain smooth temporal transitions. A Hamming window was chosen to suppress spectral leakage at frame boundaries, which is defined by the standard formula:

(1) $$w\left(n\right)=0.54-0.46\cos\left(\frac{2\pi n}{N-1}\right),\;0\leq n\leq N-1$$

where *N* denotes the frame length. This window function is a classical definition in digital signal processing [19]. These parameters were applied to MFCCs, mel spectrograms, and spectral contrast to suppress spectral leakage introduced by framing and ensure comparability in time–frequency resolution.

This augmentation strategy aims to constrain the model’s reliance on the specific background acoustic environment of the source domain, thereby enhancing robustness to background variation and providing a more stable feature foundation for subsequent cross-apiary transfer learning. Specifically, 2000 independently recorded motorcycle noise samples from the BAD dataset were superimposed onto the original audio with random SNRs ranging from −5 dB to 10 dB. Motorcycle noise was chosen because, for beehives located in urban or roadside settings, it represents a common type of environmental interference; moreover, its broad spectral coverage substantially enriches the sample space, forcing the model to learn robust acoustic features rather than relying on specific noise patterns. The lower bound of −5 dB forces the model to extract effective features even when the target signal is nearly overwhelmed; the upper bound of 10 dB ensures that the model can still capture essential target patterns under perturbation, preventing overfitting to noise.

The signal-to-noise ratio is defined as:

(2) $$\mathrm{SNR}=10\log_{10}\left(\frac{\sum_{i}x^{2}\left(i\right)}{\sum_{i}\eta^{2}\left(i\right)}\right)$$

where x(i) denotes the clean bee sound signal and η(i) denotes the motorcycle noise signal.

Two augmented versions were generated for each training sample. All samples were split at the recording-hour level to prevent data leakage, yielding 2876 queen-present and 1400 queen-absent training files. Each received two augmented copies, resulting in 5752 positive and 2800 negative segments. The final training set thus consisted exclusively of augmented samples (5752 positive and 2800 negative). Validation and test sets were kept as original clean samples without augmentation to ensure realistic evaluation.

**Figure 1:**
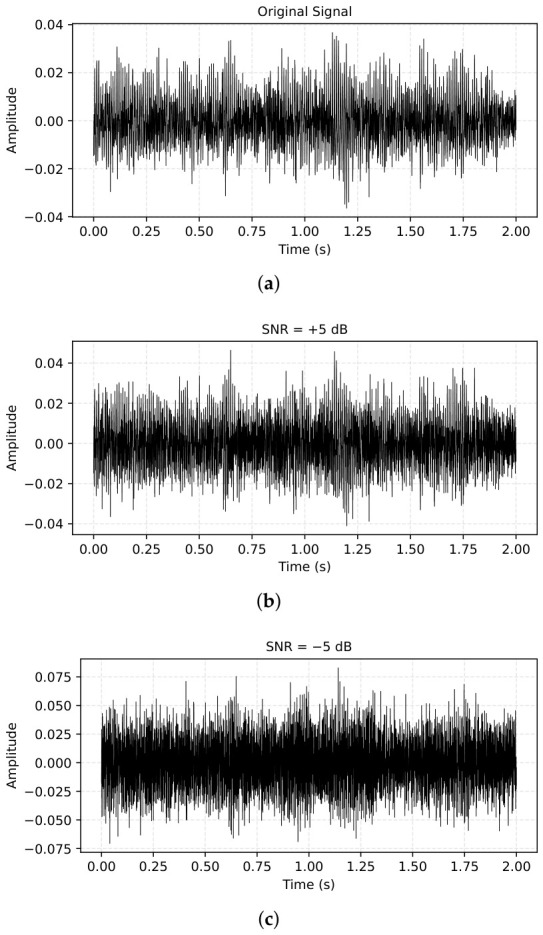
(**a**) Original clean signal (first 2 s). (**b**) Signal after motorcycle noise superposition with SNR = 5 dB. (**c**) Signal after motorcycle noise superposition with SNR = −5 dB.

Figure 1 compares the original clean signal and the signals after motorcycle noise superposition under different SNR conditions (with a 2 s segment shown). At SNR = 5 dB, the waveform remains relatively close to that of the original signal, indicating a limited degree of noise interference. At SNR = −5 dB, the amplitude of the background noise increases markedly, and the original target signal is largely overwhelmed by the noise. This comparison intuitively illustrates the range of noise intensities covered by the chosen SNR interval (−5 dB to 10 dB), with the −5 dB condition simulating extreme high-noise scenarios, intended to enhance model robustness under adverse conditions.

### 2.3. Comparative Experiments for Optimal Feature–Model Combination

**Figure 2:**
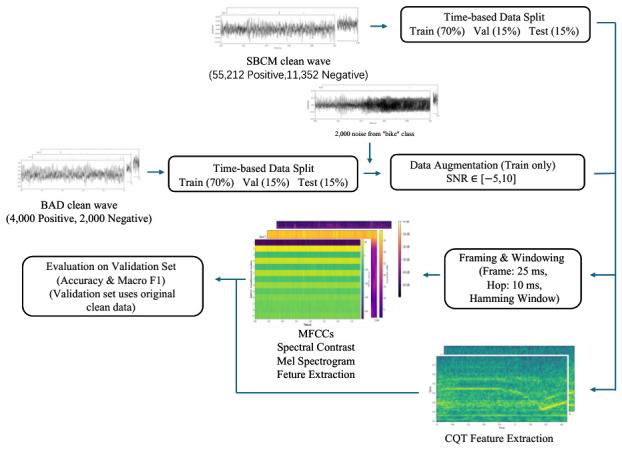
Flowchart of feature and model comparison experiments.

To identify the optimal feature–model combination, four acoustic features and five classifiers were systematically compared, as summarized in Figure 2. MFCCs, mel spectrogram, spectral contrast, and CQT were selected for comparative experiments. For classification, five models (RF, SVM, XGBoost, LR, and CNN) were selected. The aim was to establish the optimal feature–model combination as a baseline for subsequent experiments.

#### 2.3.1. Feature Extraction Methods

1. **MFCCs.** MFCCs are feature values that are widely used in audio/signal processing [15]. They are extracted by applying cepstral analysis to the mel-scale spectrum. This process involves grouping the spectrum into constant frequency bands, followed by a logarithmic transformation and an inverse fast Fourier transform. This procedure separates the correlations caused by overlapping filter banks and creates a diagonal covariance matrix, retaining only the coefficients that carry significant information. MFCCs are robust against rapid signal variations and, due to their effectiveness in capturing timbral texture, have been widely applied to beehive sound classification [12,15].
2. **Mel Spectrogram.** The mel spectrogram is a feature representation that converts sound signals into image form and is commonly used in sound analysis [20]. The audio signal is first divided into frames, and a short-time Fourier transform (STFT) is applied to obtain the spectrum for each frame. These spectra are then mapped onto the mel scale—a perceptual scale that approximates human auditory response. The resulting mel spectrogram intuitively displays the time–frequency energy distribution, with amplitude differences represented by color variations. This representation is particularly valuable for beehive status recognition, especially when used as input to convolutional neural networks [21].
3. **Spectral Contrast.** Spectral contrast captures the relative distribution of spectral peaks and valleys, effectively distinguishing harmonic components from noise in audio signals [12]. Unlike traditional spectral features that average energy across frequency bands, spectral contrast considers the difference between peaks and valleys within each sub-band, making it especially sensitive to harmonic structures and background interference. This property is particularly important in beehive monitoring, where target signals often exhibit clear harmonic patterns against environmental noise [12,13].
4. **Constant-Q Transform (CQT).** In this study, the constant-Q transform (CQT) is selected as the core acoustic feature for transfer learning. Compared with the short-time Fourier transform, the CQT provides higher frequency resolution on a logarithmic scale: its center frequencies are distributed exponentially, and the bandwidth is proportional to the center frequency, which better matches the harmonic structure of bioacoustic signals [14,22]. The CQT of a discrete-time signal x(n) is defined as:
  (3) $$X\left[k\right]=\frac{1}{N_{k}}\sum_{n=0}^{N_{k}-1}x\left(n\right)\;w\left(n\right)\;\exp\;\left(-i2\pi n\frac{f_{k}}{f_{s}}\right)$$
  where *k* is the frequency bin index; $f_{k}=f_{\min}\cdot 2^{(k-1)/B}$ is the center frequency of the *k*-th frequency band, with *B* being the number of bands per octave; $N_{k}=\left\lceil Q\cdot f_{s}/f_{k}\right\rceil$ is the adaptive window length; $Q=f_{k}/\Delta f_{k}$ is the quality factor, which remains constant across all frequency bins (in this study, Q≈17); $f_{s}$ is the sampling rate; and w(n) is the window function. In this study, the parameters are set as $f_{\min}=32$ Hz, $f_{s}=16,000$ Hz, B=12 (bins per octave), $n_{\mathrm{bins}}=84$, Q≈17, and hop_length=512.

#### 2.3.2. Classification Models

1. **Random Forest.** Random Forest (RF) [23] is an ensemble learning algorithm that constructs multiple decision trees and combines their predictions for classification purposes [21]. It exhibits strong resistance to overfitting and is effective at handling high-dimensional data. Its widespread use in studies related to beehive sound classification makes it an ideal baseline approach for this research.
2. **Support Vector Machine.** The support vector machine (SVM) maps input data into a high-dimensional space using a kernel function and seeks the optimal hyperplane that maximizes the class margin [24]. It performs well on small- to medium-sized datasets and is particularly suitable for nonlinear classification problems. Nolasco et al. [9,15] employed SVM combined with MFCC features in queen status recognition tasks and achieved promising results. As a kernel method widely applied in acoustic classification, SVM allows this study to examine how such approaches compare with tree-based and deep learning models on this task.
3. **XGBoost.** Extreme Gradient Boosting (XGBoost) is an optimized boosting algorithm built on the gradient boosting framework [25], which iteratively trains weak classifiers and combines them with weighted contributions while regularization terms control model complexity. Its consistent strong performance on structured features complements Random Forest, together offering a more complete picture of tree-based methods.
4. **Logistic Regression.** Logistic regression (LR) maps a linear combination of features to class probabilities via the sigmoid function, offering good interpretability through its simple structure. A linear baseline of this kind provides a reference point against which the gains of more complex models can be assessed.
5. **Convolutional Neural Network.** Convolutional neural networks (CNNs) learn hierarchical representations from time–frequency inputs through stacked convolutional, pooling, and fully connected layers, with local connectivity and weight sharing enabling the extraction of translation-invariant deep features. Their architecture lends itself naturally to transfer learning: weights pre-trained on a source domain can be rapidly adapted to a target domain via fine-tuning. This property motivates the choice of a lightweight shallow CNN as the deep learning representative in the comparison.

Spanning linear baselines, tree-based models, kernel methods, and deep learning, these five model types together enable a comprehensive comparison across mainstream algorithmic paradigms. By systematically evaluating their performance on different acoustic features, this study aims to identify the optimal feature–model combination for subsequent cross-apiary transfer learning experiments.

### 2.4. Ablation Experiments and Transfer Learning

#### 2.4.1. Overall Framework

To determine the optimal convolutional neural network architecture for queen status recognition and evaluate its cross-domain generalization ability, ablation experiments and transfer learning are integrated within a single experimental framework. The source domain BAD is used for model pre-training, and the target domain SBCM is used for evaluation. The ablation experiments proceed in multiple rounds: full-variable ablation is first conducted to identify the optimal architecture and hyperparameters, followed by exploration of loss functions and post-processing strategies, and comparison of learning rate strategies, finally systematic comparison of four transfer strategies using the selected optimal configuration. Accuracy, macro-averaged F1-score, AUC, negative-class F1-score, and negative-class recall are recorded for each model to provide a comprehensive assessment of performance.

In the early ablation rounds, approximately one-tenth of the target-domain training set is randomly sampled as a training subset, yielding 6332 segments (5000 positive, 1332 negative). Correspondingly, the validation and test sets were proportionally reduced to match this scale. This reduced scale lowers the computational cost of screening a large number of configurations, while also simulating a scenario in which only limited labeled target-domain data are available, thereby testing the effectiveness of transfer learning under few-sample conditions. The final transfer strategy comparison uses the full target-domain training set of 46,668 segments to evaluate performance upper bounds with abundant data. All ablation evaluations are conducted on the target-domain validation set, with the source domain used solely for pre-training.

**Figure 3:**
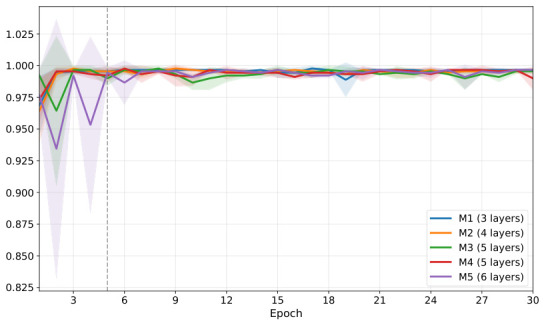
Convergence curves of negative-class recall for five CNN architectures on the source domain BAD. Different colors represent different architectures: M1 (3 layers), M2 (4 layers asymmetric), M3 (5 layers symmetric), M4 (5 layers asymmetric), and M5 (6 layers).

Figure 3 presents the convergence curves of negative-class recall (with model selection guided by negative-class F1-score) over training epochs for five common CNN architectures on the source domain BAD. All architectures stabilize by epoch 5, indicating that the classification task on this dataset is relatively simple and that five epochs are sufficient for feature learning. Accordingly, the number of pre-training epochs is fixed at 5 throughout this study.

#### 2.4.2. Full-Variable Ablation Experiments

Full-variable ablation is conducted on the target-domain validation set across five candidate architectures, all of which are purely convolutional:

1. M1: 3 convolutional layers, channels [16, 32, 64], symmetric 3 × 3 kernels;
2. M2: 4 convolutional layers, channels [16, 32, 64, 128], alternating 3 × 3 and 3 × 1 kernels;
3. M3: 5 convolutional layers, channels [16, 32, 64, 128, 128], symmetric 3 × 3 kernels;
4. M4: 5 convolutional layers, channels [16, 32, 64, 128, 128], alternating 3 × 3 and 3 × 1 kernels;
5. M5: 6 convolutional layers, channels [16, 32, 64, 128, 128, 128], symmetric 3 × 3 kernels.

The variable space covers pooling method (2 × 2 max pooling, 3 × 3 max pooling, 2 × 2 average pooling, 3 × 3 average pooling), batch normalization (enabled or disabled), and dropout rate (0.3, 0.5, 0.7), yielding 120 combinations, each repeated 3 times.

The ablation is performed twice, using negative-class recall and negative-class F1-score as the selection metric, respectively, to investigate the influence of different primary metrics on model selection. The complete results of both runs are provided in the Appendix A.

#### 2.4.3. Loss Function and Learning Rate Strategies

Building upon the configurations selected in the previous round, we further explored strategies for addressing class imbalance. For the loss function, the variable space includes class weighting (negative-class weights of 1 as baseline, 3, and 5), Focal Loss (γ=2), and threshold tuning.

To examine training stability and convergence completeness, three learning rate strategies were compared: a constant low rate ($1\times 10^{-5}$), a constant high rate ($3\times 10^{-4}$), and a sweep strategy ($1\times 10^{-5}$, switching to $3\times 10^{-4}$ at epoch 15). Each strategy was repeated three times. All evaluations used the negative-class F1-score on the target domain.

#### 2.4.4. Transfer Strategy Comparison

The three best candidate models from the previous rounds are each repeated three times on the full target-domain training set (46,668 segments). The optimal model is then used to compare four transfer strategies:

1. Direct testing: all parameters of the source-domain pre-trained model are frozen, and inference is performed directly on the target-domain test set.
2. Feature extraction: the convolutional layers are frozen, and only the fully connected classifier is trained on the target-domain training set.
3. Fine-tuning: all parameters are updated with a reduced learning rate to adapt the source-domain pre-trained model to the target domain.
4. Training from scratch: the model is randomly initialized and trained on the target-domain training set without using pre-trained weights, serving as a baseline.

Each strategy is run for 40 epochs.

### 2.5. Visualization Methods

To examine the effect of noise augmentation on sample distribution, t-distributed Stochastic Neighbor Embedding (t-SNE) was applied to the CQT features of the source domain (BAD). A total of 500 positive and 500 negative samples were randomly selected from the training set, with both original and augmented samples included. The flattened CQT spectrograms served as input, and the t-SNE algorithm projected the data into two-dimensional and three-dimensional spaces for visualization (perplexity = 30, iterations = 1000). The X and Y axes in the resulting plots represent abstract dimensions after dimensionality reduction and do not correspond to specific physical quantities; the spatial distance between any two points reflects their similarity in the original high-dimensional feature space.

**Figure 4:**
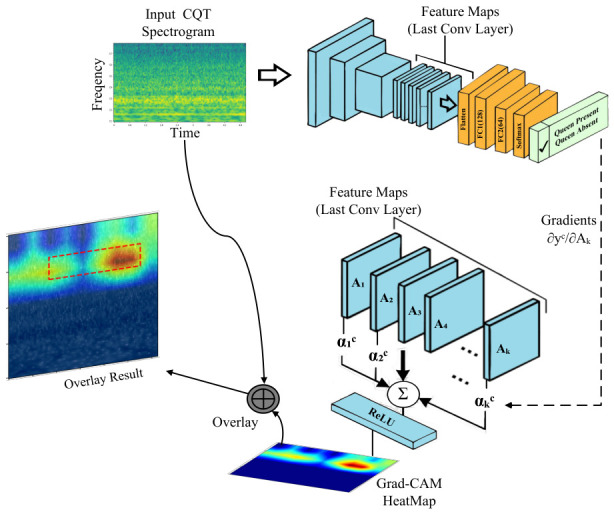
Flowchart of the Grad-CAM computation pipeline with the example of the queen-absent class.

To visually compare the transfer of source-domain pre-trained features to the target domain, Gradient-weighted Class Activation Mapping (Grad-CAM), a technique already applied in beehive acoustics research [14,26,27], was employed on the last convolutional layer of the optimal CNN model. The full pipeline is shown in Figure 4. The input is a CQT spectrogram, where the horizontal axis corresponds to time frames and the vertical axis to log-frequency. The CNN outputs two classes: queen-present (c=1) and queen-absent (c=0); the flowchart illustrates the procedure using a queen-absent example. For a given input, let $y^{c}$ denote the predicted score for the queen-absent class (c=0), and let $A^{k}$ be the *k*-th feature map of the last convolutional layer. The gradient of $y^{c}$ with respect to $A^{k}$ is obtained by backpropagation and globally average-pooled to yield the importance weight $\alpha_{k}^{c}$ of the *k*-th feature map for the queen-absent class:

(4) $$\alpha_{k}^{c}=\frac{1}{Z}\sum_{i}\sum_{j}\frac{\partial y^{c}}{\partial A_{ij}^{k}}$$

where *Z* is the number of pixels in the feature map and $A_{ij}^{k}$ is the activation at position (i,j). The weights are then used in a weighted summation of the feature maps, followed by a ReLU activation to produce a coarse heatmap. The final localization map is

(5) $$L^{c}=\mathrm{ReLU}\left(\sum_{k}\alpha_{k}^{c}A^{k}\right).$$

This heatmap is upsampled and overlaid onto the original CQT spectrogram. By comparing the similarity of highlighted regions across frequency bands between source and target domains, one can observe whether the time–frequency features learned from the source domain have been transferred to the target domain.

## 3. Results

### 3.1. Feature and Model Comparison Results

From Table 1, it can be observed that the classification difficulty of the source domain (BAD) is relatively low, with various feature-model combinations achieving accuracy and F1-scores exceeding 95%. This supports our judgment that BAD is suitable as a pre-training source domain. In contrast, the acoustic environment of the target domain (SBCM) is more complex and the recognition task is more challenging. The best performance on SBCM is achieved by the combination of Mel features and the CNN model, reaching an accuracy of 91.15% and a macro-average F1-score of 0.821 on the test set. Although the amount of data is sufficient, its performance remains significantly lower than that on the source domain.

Further observation reveals that the combination of CQT features and the CNN model exhibits stable recognition performance on both datasets, with cross-domain consistency superior to other combinations. This cross-domain stability, together with the inherent suitability of CNNs for transfer learning through fine-tuning, motivates the selection of CQT + CNN as the baseline architecture for the subsequent transfer learning experiments.

### 3.2. Ablation Experiments and Training Hyperparameters

#### 3.2.1. Metric Selection and Configuration Analysis

Appendix Table A1 presents the results of 120 configurations evaluated with negative-class recall as the selection metric. Among them, the configuration with the highest negative-class recall—M5 with 2 × 2 average pooling, batch normalization enabled, and dropout 0.7—achieved a negative-class recall mean of 97.22% but a negative-class F1 mean of only 41.28%. This indicates that the model sacrificed precision heavily by misclassifying a large number of positive samples as negative. Even the best negative-class F1 among the configurations selected under the recall criterion—M3 with 2 × 2 max pooling, batch normalization disabled, and dropout 0.5—reached only a negative-class F1 mean of 65.89%, showing an inability to balance precision and recall.

Appendix Table A2 reports the results of the same 120 combinations re-evaluated with negative-class F1 as the selection metric. The top configuration—M3 with 2 × 2 max pooling, batch normalization disabled, and dropout 0.7—attained a negative-class F1 mean of 71.23% and a negative-class recall mean of 70.89%, demonstrating a considerably better balance between the two objectives. Negative-class F1 was therefore adopted as the primary selection metric for all subsequent experiments.

**Figure 5:**
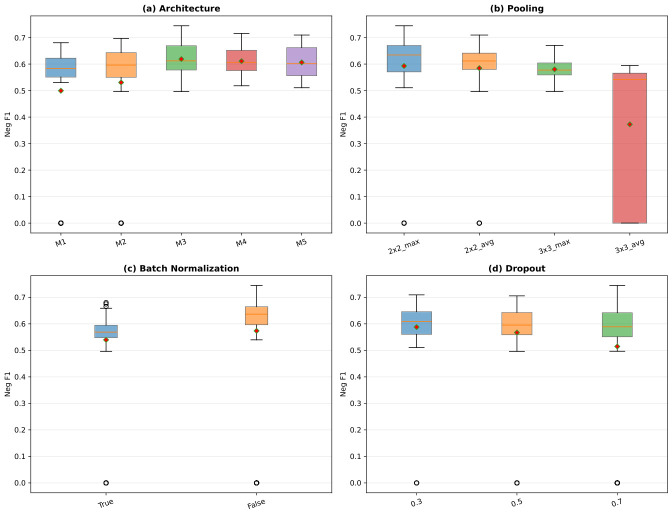
Effects of architecture, pooling method, batch normalization, and dropout on the negative-class F1 score. In each box, the colored block spans the interquartile range, the internal red line marks the median, the red diamond indicates the mean, and the whiskers extend to non-outlier extremes; small black circles denote outliers.

Figure 5 uses box plots to examine how architecture, pooling method, batch normalization, and dropout affect the negative-class F1 mean. In each box, the colored block spans the interquartile range, the internal red line marks the median, the red diamond indicates the mean, and the whiskers extend to non-outlier extremes; small black circles denote outliers.

Among the five architectures, M3 (5 layers, symmetric 3 × 3 kernels) performed best with a mean F1 of 0.71 and a variance of 0.03. M4 and M5 ranked next, while M1 and M2 were lower. The five-layer design achieved a favorable trade-off between representation capacity and overfitting risk. For pooling, 2 × 2 max pooling outperformed the other three methods, and all Top 15 configurations adopted it. The 3 × 3 pooling was viable only for M1 (3 layers) and was excluded for deeper architectures due to feature map collapse. Regarding batch normalization, the disabled condition yielded a mean F1 of 0.54, noticeably higher than the 0.47 obtained with BN enabled. The BN statistics learned during source-domain pre-training were not suitable for the target domain, and disabling BN improved domain adaptation. Dropout rates between 0.3 and 0.7 produced no significant difference and constituted a non-decisive variable.

#### 3.2.2. Loss Function and Learning Rate Strategies

After the optimal architecture was determined, class imbalance was addressed by testing 12 combinations of class weights (1, 3, 5), Focal Loss (γ=2), and threshold tuning on five candidate configurations, each repeated three times. Appendix Table A3 summarizes the negative-class F1 scores. Threshold tuning was the only strategy that yielded consistent improvement; class weighting and Focal Loss did not surpass the unweighted baseline.

Table 2 compares three learning rate strategies. With the low learning rate ($1\times 10^{-5}$), the gradient updates were too small to effectively distinguish negative samples, and the negative-class F1 remained near zero. The early-stopping mechanism was triggered at epoch 1, which was recorded as the best epoch. The sweep strategy ($1\times 10^{-5}$ switching to $3\times 10^{-4}$ at epoch 10) converged but underperformed for the constant high learning rate. The constant high learning rate ($3\times 10^{-4}$) achieved the highest negative-class F1 (0.72) and overall accuracy, and was adopted for the final full-data experiment.

## 4. Discussion

### 4.1. Transfer Strategy Comparison

**Figure 6:**
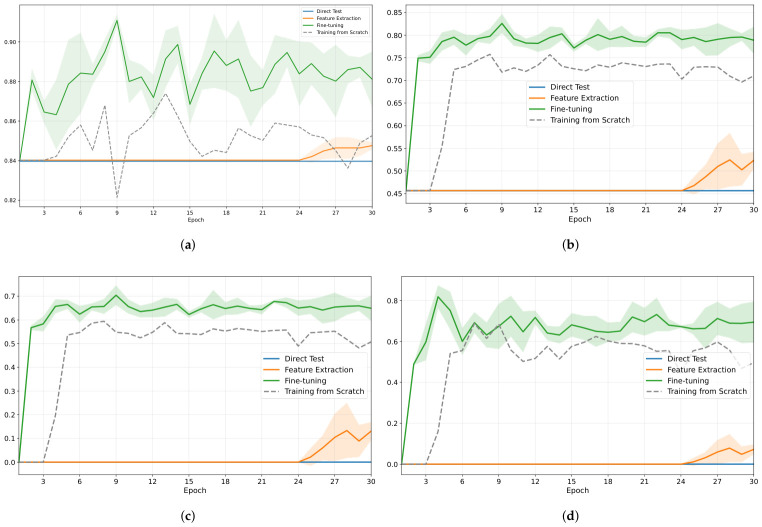
Performance of four transfer strategies on the small training subset (approximately 6000 samples). (**a**) Accuracy, (**b**) macro F1, (**c**) negative-class F1, (**d**) negative-class recall. Shaded regions indicate ±1 standard deviation across three independent runs.

Figure 6 compares the four transfer strategies on the small training subset using M3 (2 × 2 max pooling, BN = False, dropout 0.7), the best-performing configuration in the small-sample experiments. Training from scratch serves as the baseline. Direct testing yielded the lowest results across all metrics; negative-class F1 and negative-class recall both remained at zero, indicating that no effective features were learned. Feature extraction improved gradually but remained limited. Fine-tuning achieved the best results across all metrics and the fastest convergence rate.

To examine the upper bound of model performance, the three best-performing candidate configurations from the ablation experiments—namely M3 with 2 × 2 max pooling, BN disabled, and dropout 0.5, M3 with 2 × 2 max pooling, BN disabled, and dropout 0.7, and M5 with 2 × 2 max pooling, BN disabled, and dropout 0.3—were fine-tuned on the full target-domain training set of 46,668 segments, each with three independent repeats. Table 3 summarizes the results. The three candidates achieved comparable performance; M3 with dropout 0.5 was selected as the optimal model owing to its smallest standard deviation.

**Figure 7:**
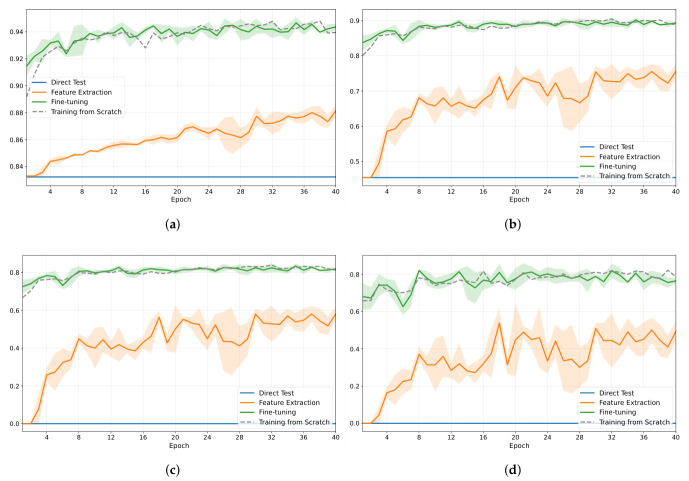
Performance of four transfer strategies on the full target-domain training set (46,668 segments). (**a**) Accuracy, (**b**) macro F1, (**c**) negative-class F1, (**d**) negative-class recall. Shaded regions indicate ±1 standard deviation across three independent runs.

Figure 7 compares the four transfer strategies on the full target-domain training set.Under the full-data setting, direct testing remained unable to identify negative samples. Feature extraction gained moderate detection ability but still fell considerably below fine-tuning and training from scratch. Fine-tuning and training from scratch achieved comparable final performance; fine-tuning exhibited a faster convergence rate in the early training stage.

**Figure 8:**
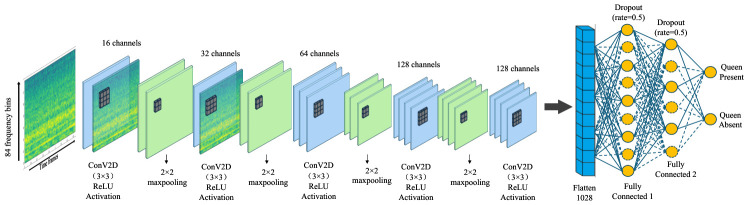
The overall architecture of BeeNet.

The architecture of the final model, named BeeNet, is illustrated in Figure 8. The input is a CQT spectrogram of size 1 × 84 × 156. Five convolutional layers with 3 × 3 kernels (channel sizes 16, 32, 64, 128, 128) are applied, each followed by ReLU activation and 2 × 2 max pooling. The feature maps are then flattened into a 1024-dimensional vector and passed through two fully connected layers (1024→128→64) with ReLU activation and dropout 0.5. The output layer produces the binary prediction: queen present or queen absent.

### 4.2. The Role of t-SNE Visualization Noise on Sample Distribution

**Figure 9:**
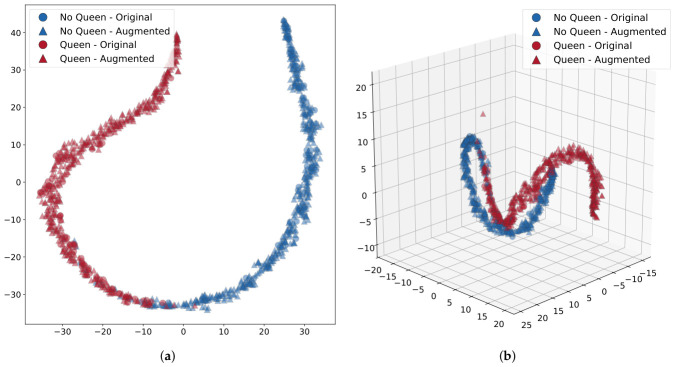
t-SNE visualization results: (**a**) 2D projection; (**b**) 3D projection.

Figure 9 presents the t-SNE projection of CQT features from the source domain. Positive and negative samples form clearly separated clusters, confirming that CQT features can effectively discriminate between queen presence and absence. Augmented samples (triangles) are distributed around the original samples (circles), filling sparse regions of the original distribution and extending toward the class boundary. This pattern indicates that noise augmentation expands the sample coverage while preserving class separability, thereby supplying a richer and more stable feature basis for the subsequent transfer learning.

### 4.3. Grad-CAM Visualization and Validation of Transfer Learning Effectiveness

**Figure 10:**
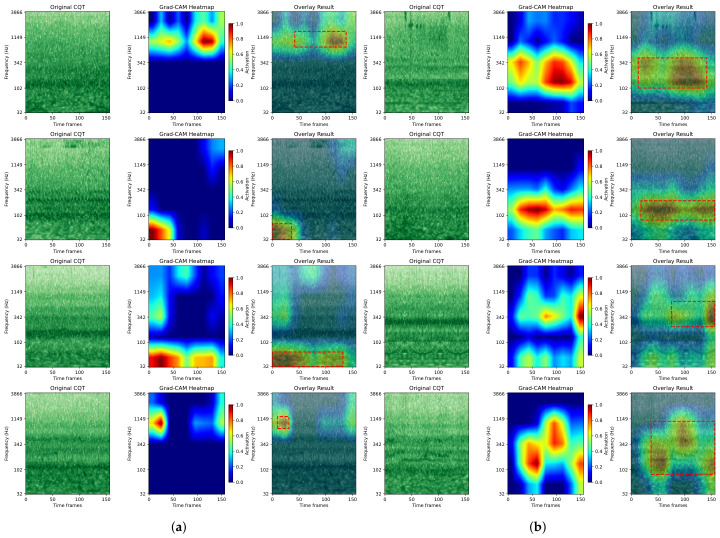
Grad-CAM visualization comparison: (**a**) queen-present (positive) samples in BAD and SBCM; (**b**) queen-loss (negative) samples in BAD and SBCM. Within each subfigure, the top two rows originate from the source domain (BAD) and the bottom two rows from the target domain (SBCM). In the heatmaps, red and yellow regions indicate high activation, while blue regions indicate low activation. Red dashed boxes mark regions where the heatmap value exceeds 0.7 times the maximum.

Figure 10 shows Grad-CAM heatmaps for positive and negative samples. For each class, four samples are displayed: the first two rows originate from the source domain (BAD) and the last two rows from the target domain (SBCM). Every row corresponds to one sample and consists of three columns—the original CQT spectrogram, the Grad-CAM heatmap, and their overlay. Red dashed boxes mark regions where the heatmap value exceeds 0.7 times the maximum.

For positive samples, the highlighted regions are concentrated and occupy stable frequency bands, suggesting that the model relies on consistent local time–frequency features. For negative samples, the highlighted regions are dispersed and cover a wide frequency range, reflecting the absence of a consistent acoustic pattern under queen-absent conditions. The frequency bands highlighted in the source and target domains display clear similarity, indicating that the time–frequency features learned from the source domain are effectively transferred to the target domain. This visual evidence supports the effectiveness of the transfer learning strategy from the perspective of feature focus.

### 4.4. Final Evaluation of BeeNet Under Few-Sample and Full-Data Conditions

To evaluate the effectiveness of the proposed model architecture and training strategy, BeeNet (M3 with 2 × 2 max pooling, batch normalization disabled, and dropout rate 0.5) was assessed under two target-domain data regimes: (1) a few-sample setting using approximately 10% of the SBCM training data (6332 segments, positive-to-negative ratio ≈ 3.75:1), and (2) a full-data setting using the entire training set (46,668 segments, positive-to-negative ratio ≈ 4.9:1). After training, the model was evaluated on an independent, held-out test set comprising 9984 segments.

Table 4 reports the final test set performance of BeeNet under the two data conditions. The full-data model outperforms the few-sample model across all metrics; nonetheless, the few-sample model still achieves competitive results, with a negative-class F1-score near 0.79 and a negative-class recall near 0.78.

**Figure 11:**
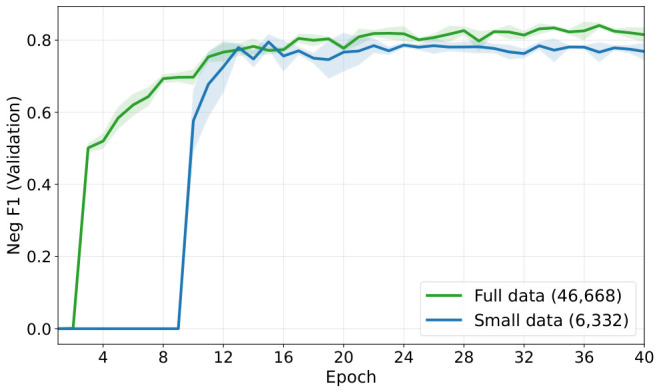
Convergence curves of negative-class F1-score on the complete target-domain validation set for BeeNet under few-sample and full-data settings. Values are reported as mean over three independent runs. Shaded regions indicate ±1 standard deviation across three independent runs.

Figure 11 compares the negative-class F1-score on the complete target-domain validation set as a function of fine-tuning epochs for both settings, with curves averaged over three independent runs. Under full-data conditions, the negative-class F1-score rises rapidly from zero starting at epoch 2, while under few-sample conditions it remains at zero for the first eight epochs and only begins to increase around epoch 9–10, indicating a considerably slower convergence rate. Moreover, the convergence speed of the few-sample setting on the full validation set is lower than that observed previously on the smaller validation subset used in ablation, reflecting the increased difficulty when a larger and more diverse validation set is employed. In both regimes, the curves stabilize in the later training stage without obvious declines. Once converged, the full-data model maintains a negative-class F1-score around 0.84 on the validation set, whereas the few-sample model reaches approximately 0.80.

## 5. Conclusions

This study addressed the generalization challenge in acoustic-based queen status recognition across apiaries through data augmentation, architecture optimization, and transfer learning. At the data level, motorcycle noise augmentation with random signal-to-noise ratios was applied to the source-domain audio. t-SNE visualization confirmed that augmented samples expanded the sample coverage while preserving class separability, thereby supplying a richer and more stable feature basis for the subsequent transfer learning. At the model and training hyperparameter level, negative-class F1 on the target domain via fine-tuning was established as the primary selection metric. A multi-round progressive ablation was conducted to determine the optimal configuration across architecture, pooling method, batch normalization, dropout rate, loss function, and learning rate. At the transfer strategy level, fine-tuning substantially outperformed feature extraction and training from scratch under few-sample conditions in both performance metrics and convergence rate, offering an effective approach for the class-imbalanced, small-sample scenario of queen absence detection. Under sufficient data, fine-tuning converged faster in the early training stage, helping reduce training cost. Grad-CAM visualization further demonstrated the similarity of transferred features between source and target domains from the perspective of feature focus. The final model, named BeeNet (M3: 5 layers, symmetric 3×3 kernels, 2×2 max pooling, batch normalization disabled, dropout 0.5), achieved an accuracy of 95.05%, a macro F1-score of 0.9148, a negative-class F1-score of 0.8596, a negative-class recall of 0.8733, and an AUC of 0.9766 on the full target-domain test set.

Despite the promising results, this study has several limitations. The experiments were conducted on only two public datasets (BAD and SBCM), and the generalization capability to more diverse apiaries with different bee species, climatic conditions, or seasonal variations remains to be evaluated. The noise augmentation strategy relied solely on motorcycle noise, and the model’s robustness against other common environmental interferences has not been verified.

Future work can evaluate queen status across different bee species in apiaries covering diverse geographic regions and acoustic interference conditions; integrate environmental parameters such as temperature and humidity for multimodal monitoring to investigate how hive microclimate modulates queen-related acoustic expressions, and extend the method to finer-grained colony status recognition, capturing long-term dynamic changes in beehive acoustics to enable early prediction of gradual colony deterioration or queen loss events.

## Figures and Tables

**Table 1:** Performance comparison of feature–model combinations on source (BAD) and target (SBCM) domains.

Model	Feature	BAD		SBCM	
		Accuracy (%)	Micro F1 (%)	Accuracy (%)	Micro F1 (%)
LR	MFCC	98.1 ± 0.1	98.0 ± 0.2	77.5 ± 0.0	66.2 ± 0.0
	Mel	93.9 ± 1.3	93.5 ± 1.3	81.3 ± 0.0	65.1 ± 0.0
	Spectral contrast	83.9 ± 0.8	83.4 ± 0.8	72.5 ± 0.0	64.2 ± 0.0
	CQT	91.1 ± 0.7	90.7 ± 0.7	76.0 ± 0.0	62.1 ± 0.0
SVM	MFCC	92.9 ± 0.5	92.5 ± 0.5	90.3 ± 0.0	80.5 ± 0.0
	Mel	98.4 ± 0.5	98.3 ± 0.6	90.2 ± 0.0	79.2 ± 0.0
	Spectral contrast	85.8 ± 0.2	85.3 ± 0.2	81.8 ± 0.0	69.5 ± 0.0
	CQT	99.1 ± 0.4	99.0 ± 0.4	89.0 ± 0.0	76.1 ± 0.0
RF	MFCC	95.0 ± 1.0	94.5 ± 1.1	90.5 ± 0.5	78.0 ± 1.6
	Mel	95.3 ± 0.8	95.0 ± 0.9	88.7 ± 0.4	72.2 ± 1.6
	Spectral contrast	91.2 ± 0.2	90.5 ± 0.2	86.7 ± 0.2	70.1 ± 0.5
	CQT	87.6 ± 1.5	87.2 ± 1.5	87.0 ± 0.6	67.2 ± 1.8
XGBoost	MFCC	94.9 ± 1.0	94.5 ± 1.0	89.4 ± 0.0	76.0 ± 0.0
	Mel	96.4 ± 0.3	96.1 ± 0.3	89.8 ± 0.0	76.9 ± 0.0
	Spectral contrast	90.3 ± 0.6	89.7 ± 0.7	85.9 ± 0.0	69.3 ± 0.0
	CQT	98.2 ± 0.6	98.1 ± 0.6	89.7 ± 0.0	76.0 ± 0.0
CNN	MFCC	99.6 ± 0.2	99.5 ± 0.3	89.4 ± 0.8	77.3 ± 4.3
	Mel	92.7 ± 1.7	91.6 ± 1.9	91.1 ± 1.4	82.1 ± 2.9
	Spectral contrast	90.5 ± 2.1	88.1 ± 2.3	84.8 ± 0.4	68.1 ± 1.1
	CQT	96.1 ± 4.4	95.5 ± 5.0	89.3 ± 0.8	79.1 ± 1.9

Values are reported as mean ± standard deviation over three independent runs. All values are rounded to one decimal place.

**Table 2:** Learning rate strategy comparison on the target-domain validation set.

LR	BE	Acc (%)	MF1 (%)	NF1 (%)	NR (%)	MR (%)	AUC (%)
low	1	15.97 ± 0.00	13.77 ± 0.00	27.54 ± 0.00	100.00 ± 0.00	50.00 ± 0.00	52.59 ± 0.09
sweep	19.33 ± 2.08	86.84 ± 0.09	79.03 ± 0.27	66.23 ± 0.60	80.80 ± 2.51	84.40 ± 0.98	88.65 ± 0.45
high	8.67 ± 0.58	90.99 ± 0.93	83.34 ± 1.91	72.05 ± 3.32	72.83 ± 4.99	83.63 ± 2.44	88.39 ± 0.41

Values are reported as mean ± standard deviation over three independent runs. LR strategies: low = $1\times 10^{-5}$; sweep = $1\times 10^{-5}$ → $3\times 10^{-4}$ at epoch 10; high = $3\times 10^{-4}$. BE: best epoch. Acc: Accuracy; MF1: Macro F1; NF1: Negative F1; NR: Negative Recall; MR: Macro Recall; AUC: Area Under the Curve.

**Table 3:** Performance of the three candidate configurations on the full target-domain validation set. Values are mean ± std over three runs.

Config	Acc (%)	MF1 (%)	NF1 (%)	NR (%)	MR (%)	AUC (%)
M3_dp = 0.5	94.61 ± 0.18	90.06 ± 0.28	83.33 ± 0.46	80.60 ± 0.31	89.01 ± 0.17	97.10 ± 0.35
M3_dp = 0.7	94.50 ± 0.49	89.91 ± 0.79	83.10 ± 1.28	80.84 ± 1.54	89.04 ± 0.70	96.69 ± 0.62
M5_dp = 0.3	94.62 ± 0.25	90.20 ± 0.56	83.62 ± 1.01	82.29 ± 2.72	89.69 ± 1.20	97.43 ± 0.40

Acc: Accuracy; MF1: Macro F1; NF1: Negative F1; NR: Negative Recall; MR: Macro Recall; AUC: Area Under the Curve. Values are reported as mean ± standard deviation.

**Table 4:** Performance of BeeNet on the independent target-domain test set under few-sample and full-data conditions.

Setting	Acc (%)	Macro F1	Neg F1	Neg Recall	Macro Recall	AUC
Few-sample	92.79 ± 0.64	0.8733 ± 0.0083	0.7900 ± 0.0127	0.7834 ± 0.0246	0.8707 ± 0.0082	0.9572 ± 0.0009
Full-data	95.05 ± 0.73	0.9148 ± 0.0117	0.8596 ± 0.0191	0.8733 ± 0.0093	0.9200 ± 0.0076	0.9766 ± 0.0032

Values are reported as mean ± standard deviation over three independent runs. Acc: Accuracy; Macro F1: macro-averaged F1-score; Neg F1: negative-class F1-score; Neg Recall: negative-class recall; Macro Recall: macro-averaged recall; AUC: area under the ROC curve.

## Data Availability

The data that support the findings of this study are openly available in two public datasets on the Kaggle platform: The “Beehive Sounds” dataset (URL: https://www.kaggle.com/datasets/annajyang/beehive-sounds, accessed on 1 June 2026). The “BeeHive Audio Dataset with Queen and without Queen” (URL: https://www.kaggle.com/datasets/harshkumar1711/beehive-audio-dataset-with-queen-and-without-queen, accessed on 1 June 2026). Both datasets are licensed under the Kaggle Terms of Service and were used in this study for source domain pre-training and target domain adaptation, respectively. The source code for data preprocessing and model training is available from the corresponding author upon reasonable request.

## Appendix A

**Table A1:** Full-variable ablation results on the target-domain validation set using negative-class recall as the selection metric. Values are mean ± std over three runs. Gray rows indicate representative configurations. NA denotes that the feature map collapsed to 0 × 0 with 3 × 3 pooling, causing a runtime error. T: Batch Normalization enabled; F: Batch Normalization disabled; DP: Dropout rate; Acc: Accuracy; MF1: Macro F1; NF1: Negative F1; NR: Negative Recall; MR: Macro Recall; AUC: Area Under the Curve.

Arch	Pooling	BN	DP	Acc (%)	MF1 (%)	NF1 (%)	NR (%)	MR (%)	AUC (%)
M1	2 × 2 Max	T	0.3	87.21 ± 1.79	78.44 ± 2.02	64.68 ± 2.96	72.95 ± 2.09	81.43 ± 0.75	88.34 ± 1.39
M1	2 × 2 Max	T	0.5	83.47 ± 0.97	55.14 ± 16.43	19.83 ± 34.33	27.05 ± 46.87	60.57 ± 18.39	63.94 ± 20.61
M1	2 × 2 Max	T	0.7	84.03 ± 0.00	45.66 ± 0.00	0.00 ± 0.00	0.00 ± 0.00	50.00 ± 0.00	49.89 ± 1.26
M1	2 × 2 Max	F	0.3	83.64 ± 1.68	74.38 ± 1.96	58.98 ± 2.79	73.43 ± 0.91	79.50 ± 1.37	87.59 ± 2.12
M1	2 × 2 Max	F	0.5	84.49 ± 3.99	75.27 ± 3.54	60.23 ± 4.46	72.58 ± 9.59	79.67 ± 2.72	88.33 ± 1.98
M1	2 × 2 Max	F	0.7	84.10 ± 1.60	75.19 ± 1.16	60.32 ± 1.16	75.46 ± 4.17	80.61 ± 0.76	87.96 ± 0.63
M1	2 × 2 Avg	T	0.3	84.99 ± 3.01	75.00 ± 3.94	59.19 ± 5.96	67.51 ± 2.82	77.91 ± 2.89	87.16 ± 1.87
M1	2 × 2 Avg	T	0.5	86.33 ± 2.38	77.39 ± 2.79	63.17 ± 4.32	73.07 ± 2.09	80.96 ± 1.98	87.38 ± 0.32
M1	2 × 2 Avg	T	0.7	84.03 ± 0.00	45.66 ± 0.00	0.00 ± 0.00	0.00 ± 0.00	50.00 ± 0.00	51.15 ± 1.35
M1	2 × 2 Avg	F	0.3	83.39 ± 0.52	73.40 ± 1.79	57.11 ± 3.35	69.57 ± 7.66	77.79 ± 3.43	87.85 ± 2.54
M1	2 × 2 Avg	F	0.5	86.00 ± 1.60	76.48 ± 2.13	61.52 ± 3.29	69.93 ± 2.86	79.49 ± 1.86	88.01 ± 1.18
M1	2 × 2 Avg	F	0.7	81.52 ± 1.89	72.15 ± 2.49	56.00 ± 5.66	73.72 ± 7.94	79.01 ± 3.79	86.61 ± 1.97
M1	3 × 3 Max	T	0.3	78.68 ± 4.86	69.39 ± 4.00	52.59 ± 4.46	72.83 ± 6.18	76.31 ± 1.45	85.79 ± 0.46
M1	3 × 3 Max	T	0.5	73.80 ± 5.96	64.97 ± 5.52	47.47 ± 5.53	72.58 ± 3.35	73.31 ± 4.03	83.37 ± 3.66
M1	3 × 3 Max	T	0.7	78.30 ± 2.07	69.40 ± 1.96	52.92 ± 2.57	76.21 ± 4.58	77.45 ± 1.95	85.71 ± 1.28
M1	3 × 3 Max	F	0.3	80.32 ± 0.93	70.06 ± 1.38	52.53 ± 2.17	68.12 ± 2.18	75.39 ± 1.44	84.99 ± 1.93
M1	3 × 3 Max	F	0.5	83.49 ± 2.45	73.30 ± 1.35	56.84 ± 0.84	67.87 ± 7.13	77.16 ± 1.61	86.33 ± 0.69
M1	3 × 3 Max	F	0.7	84.01 ± 0.52	74.60 ± 0.88	59.14 ± 1.46	72.46 ± 2.42	79.33 ± 1.24	86.97 ± 0.26
M1	3 × 3 Avg	T	0.3	79.24 ± 3.77	67.97 ± 2.61	49.01 ± 2.65	61.63 ± 5.05	72.24 ± 1.07	82.08 ± 2.29
M1	3 × 3 Avg	T	0.5	77.64 ± 4.05	67.25 ± 3.61	48.83 ± 4.06	66.06 ± 1.34	72.95 ± 2.00	84.19 ± 1.77
M1	3 × 3 Avg	T	0.7	77.53 ± 2.95	69.65 ± 1.34	52.39 ± 1.14	70.17 ± 8.05	76.41 ± 1.34	85.78 ± 0.52
M1	3 × 3 Avg	F	0.3	82.29 ± 0.96	71.68 ± 0.32	54.36 ± 0.52	66.06 ± 5.22	75.72 ± 1.23	83.45 ± 1.50
M1	3 × 3 Avg	F	0.5	81.31 ± 1.89	70.11 ± 1.43	51.86 ± 3.14	63.53 ± 11.35	74.11 ± 3.85	82.71 ± 0.30
M1	3 × 3 Avg	F	0.7	NA	NA	NA	NA	NA	NA
M2	2 × 2 Max	T	0.3	66.57 ± 13.19	60.61 ± 10.26	46.05 ± 8.51	84.90 ± 5.51	73.98 ± 6.50	82.45 ± 2.14
M2	2 × 2 Max	T	0.5	79.34 ± 0.34	70.84 ± 1.18	55.10 ± 2.26	79.59 ± 7.62	79.51 ± 3.02	83.70 ± 1.51
M2	2 × 2 Max	T	0.7	77.01 ± 9.51	69.01 ± 7.81	53.60 ± 7.91	78.99 ± 8.88	77.84 ± 2.40	84.54 ± 1.70
M2	2 × 2 Max	F	0.3	86.13 ± 0.19	76.98 ± 0.70	62.47 ± 1.69	72.46 ± 6.03	80.59 ± 2.44	86.10 ± 0.72
M2	2 × 2 Max	F	0.5	85.64 ± 3.37	74.15 ± 4.29	59.67 ± 5.57	83.89 ± 4.93	81.60 ± 2.45	85.37 ± 2.18
M2	2 × 2 Max	F	0.7	84.16 ± 0.46	76.45 ± 0.43	62.97 ± 0.62	84.30 ± 1.72	84.22 ± 0.57	86.73 ± 0.38
M2	2 × 2 Avg	T	0.3	80.69 ± 4.88	72.93 ± 3.83	58.48 ± 4.35	83.94 ± 3.88	82.10 ± 1.24	87.52 ± 0.86
M2	2 × 2 Avg	T	0.5	79.61 ± 4.85	72.18 ± 4.00	57.87 ± 4.43	86.35 ± 3.82	82.34 ± 1.29	87.50 ± 0.19
M2	2 × 2 Avg	T	0.7	78.26 ± 8.38	71.15 ± 7.25	57.03 ± 7.96	86.47 ± 5.80	81.59 ± 3.15	86.98 ± 1.08
M2	2 × 2 Avg	F	0.3	84.30 ± 2.92	75.55 ± 2.59	60.80 ± 4.50	75.64 ± 1.34	80.78 ± 1.96	87.44 ± 1.15
M2	2 × 2 Avg	F	0.5	81.50 ± 3.19	73.19 ± 2.84	58.28 ± 3.37	80.22 ± 3.23	80.97 ± 0.65	86.52 ± 0.55
M2	2 × 2 Avg	F	0.7	82.27 ± 1.99	73.97 ± 1.49	59.28 ± 1.94	80.56 ± 4.24	81.58 ± 0.93	86.58 ± 0.32
M2	3 × 3 Max	T	0.3	71.68 ± 9.25	63.55 ± 6.64	46.87 ± 4.67	75.85 ± 11.06	73.37 ± 1.99	80.81 ± 1.87
M2	3 × 3 Max	T	0.5	75.15 ± 2.86	66.58 ± 2.77	49.67 ± 3.32	76.45 ± 2.52	75.69 ± 2.41	82.95 ± 1.99
M2	3 × 3 Max	T	0.7	65.26 ± 15.78	59.55 ± 11.37	42.27 ± 9.19	84.42 ± 11.06	73.02 ± 5.29	83.01 ± 1.20
M2	3 × 3 Max	F	0.3	82.75 ± 1.99	72.26 ± 1.42	55.22 ± 1.81	66.43 ± 5.27	76.14 ± 1.40	85.56 ± 0.63
M2	3 × 3 Max	F	0.5	85.76 ± 1.07	75.89 ± 1.72	60.45 ± 2.84	68.12 ± 3.49	78.62 ± 1.77	87.80 ± 0.81
M2	3 × 3 Max	F	0.7	85.05 ± 1.25	74.69 ± 0.86	58.51 ± 0.93	65.94 ± 4.01	77.31 ± 1.01	87.21 ± 0.89
M2	3 × 3 Avg	T	0.3	72.99 ± 3.36	64.11 ± 2.94	46.28 ± 3.31	72.46 ± 2.90	72.79 ± 2.53	80.16 ± 2.51
M2	3 × 3 Avg	T	0.5	73.09 ± 6.20	64.91 ± 4.34	48.16 ± 3.41	77.08 ± 8.90	74.70 ± 1.28	82.27 ± 1.24
M2	3 × 3 Avg	T	0.7	79.71 ± 6.05	70.02 ± 4.32	53.15 ± 3.88	70.65 ± 12.49	76.04 ± 2.06	84.35 ± 0.54
M2	3 × 3 Avg	F	0.3	NA	NA	NA	NA	NA	NA
M2	3 × 3 Avg	F	0.5	NA	NA	NA	NA	NA	NA
M2	3 × 3 Avg	F	0.7	NA	NA	NA	NA	NA	NA
M3	2 × 2 Max	T	0.3	69.37 ± 6.94	63.45 ± 5.62	48.91 ± 5.37	90.10 ± 2.78	77.76 ± 2.99	87.23 ± 1.11
M3	2 × 2 Max	T	0.5	76.00 ± 2.01	68.53 ± 1.86	53.23 ± 1.84	85.27 ± 3.99	79.42 ± 1.22	85.93 ± 0.88
M3	2 × 2 Max	T	0.7	76.99 ± 3.16	69.16 ± 2.84	53.67 ± 4.04	83.21 ± 7.74	79.51 ± 3.81	87.19 ± 2.17
M3	2 × 2 Max	F	0.5	86.17 ± 1.97	78.61 ± 2.59	65.89 ± 3.97	83.45 ± 5.51	85.07 ± 2.69	88.33 ± 0.81
M3	2 × 2 Max	F	0.3	86.94 ± 1.84	78.86 ± 1.75	65.81 ± 2.29	78.38 ± 5.07	83.47 ± 2.16	88.61 ± 2.51
M3	2 × 2 Max	F	0.7	85.53 ± 0.92	77.79 ± 1.52	64.57 ± 1.78	82.94 ± 4.30	84.50 ± 2.24	87.72 ± 0.94
M3	2 × 2 Avg	T	0.3	77.39 ± 3.43	68.89 ± 2.51	52.69 ± 2.48	78.38 ± 5.81	77.79 ± 1.39	84.13 ± 0.04
M3	2 × 2 Avg	T	0.5	69.50 ± 9.98	62.14 ± 6.45	46.05 ± 3.98	79.35 ± 14.96	73.49 ± 1.95	84.09 ± 1.04
M3	2 × 2 Avg	T	0.7	76.37 ± 6.79	68.60 ± 5.59	53.13 ± 5.70	81.76 ± 5.33	78.62 ± 2.25	86.35 ± 0.55
M3	2 × 2 Avg	F	0.3	82.97 ± 4.70	74.89 ± 3.61	60.68 ± 4.69	80.56 ± 0.64	82.00 ± 2.69	85.49 ± 2.43
M3	2 × 2 Avg	F	0.5	82.39 ± 1.01	73.78 ± 0.52	58.70 ± 0.54	78.26 ± 3.37	80.72 ± 0.68	85.53 ± 0.67
M3	2 × 2 Avg	F	0.7	85.93 ± 1.51	77.84 ± 1.53	64.45 ± 1.98	79.59 ± 3.89	83.37 ± 1.22	86.91 ± 1.17
M3	3 × 3 Max	T	0.3	NA	NA	NA	NA	NA	NA
M3	3 × 3 Max	T	0.5	NA	NA	NA	NA	NA	NA
M3	3 × 3 Max	T	0.7	NA	NA	NA	NA	NA	NA
M3	3 × 3 Max	F	0.3	NA	NA	NA	NA	NA	NA
M3	3 × 3 Max	F	0.5	NA	NA	NA	NA	NA	NA
M3	3 × 3 Max	F	0.7	NA	NA	NA	NA	NA	NA
M3	3 × 3 Avg	T	0.3	NA	NA	NA	NA	NA	NA
M3	3 × 3 Avg	T	0.5	NA	NA	NA	NA	NA	NA
M3	3 × 3 Avg	T	0.7	NA	NA	NA	NA	NA	NA
M3	3 × 3 Avg	F	0.3	NA	NA	NA	NA	NA	NA
M3	3 × 3 Avg	F	0.5	NA	NA	NA	NA	NA	NA
M3	3 × 3 Avg	F	0.7	NA	NA	NA	NA	NA	NA
M4	2 × 2 Max	T	0.3	67.46 ± 2.65	61.76 ± 2.31	47.03 ± 2.35	90.22 ± 1.47	76.67 ± 1.89	85.03 ± 1.21
M4	2 × 2 Max	T	0.5	71.56 ± 9.40	63.99 ± 8.58	47.63 ± 9.98	78.14 ± 10.56	74.23 ± 8.51	78.46 ± 9.30
M4	2 × 2 Max	T	0.7	61.80 ± 13.07	56.71 ± 9.43	42.99 ± 5.81	86.84 ± 5.42	71.94 ± 4.92	83.28 ± 2.03
M4	2 × 2 Max	F	0.3	85.49 ± 1.69	76.58 ± 1.83	62.14 ± 2.55	74.28 ± 1.21	80.95 ± 0.75	86.33 ± 0.65
M4	2 × 2 Max	F	0.5	86.09 ± 3.43	78.01 ± 3.90	64.70 ± 5.39	78.74 ± 2.58	83.12 ± 2.30	86.95 ± 1.13
M4	2 × 2 Max	F	0.7	85.94 ± 1.53	77.69 ± 1.81	64.13 ± 2.59	78.50 ± 1.27	82.93 ± 1.13	87.39 ± 0.94
M4	2 × 2 Avg	T	0.3	67.44 ± 8.24	61.99 ± 6.79	47.87 ± 5.78	91.67 ± 3.72	77.25 ± 4.40	86.99 ± 3.48
M4	2 × 2 Avg	T	0.5	74.36 ± 6.18	66.05 ± 5.04	48.98 ± 4.20	86.47 ± 5.80	79.25 ± 1.29	87.84 ± 0.43
M4	2 × 2 Avg	T	0.7	72.45 ± 8.97	65.74 ± 6.97	50.91 ± 5.04	86.60 ± 7.29	78.18 ± 3.21	86.82 ± 0.91
M4	2 × 2 Avg	F	0.3	82.89 ± 2.59	74.08 ± 3.03	58.97 ± 4.39	76.57 ± 2.52	80.33 ± 2.49	88.04 ± 2.96
M4	2 × 2 Avg	F	0.5	82.79 ± 3.13	73.13 ± 4.72	57.02 ± 7.56	71.38 ± 9.33	78.17 ± 5.45	84.18 ± 5.47
M4	2 × 2 Avg	F	0.7	82.50 ± 0.41	73.14 ± 2.11	57.29 ± 4.28	74.15 ± 11.26	79.13 ± 4.74	86.84 ± 4.96
M4	3 × 3 Max	T	0.3	NA	NA	NA	NA	NA	NA
M4	3 × 3 Max	T	0.5	NA	NA	NA	NA	NA	NA
M4	3 × 3 Max	T	0.7	NA	NA	NA	NA	NA	NA
M4	3 × 3 Max	F	0.3	NA	NA	NA	NA	NA	NA
M4	3 × 3 Max	F	0.5	NA	NA	NA	NA	NA	NA
M4	3 × 3 Max	F	0.7	NA	NA	NA	NA	NA	NA
M4	3 × 3 Avg	T	0.3	NA	NA	NA	NA	NA	NA
M4	3 × 3 Avg	T	0.5	NA	NA	NA	NA	NA	NA
M4	3 × 3 Avg	T	0.7	NA	NA	NA	NA	NA	NA
M4	3 × 3 Avg	F	0.3	NA	NA	NA	NA	NA	NA
M4	3 × 3 Avg	F	0.5	NA	NA	NA	NA	NA	NA
M4	3 × 3 Avg	F	0.7	NA	NA	NA	NA	NA	NA
M5	2 × 2 Max	T	0.3	61.84 ± 2.73	57.03 ± 2.55	42.67 ± 2.69	88.77 ± 4.46	72.75 ± 3.04	83.58 ± 4.48
M5	2 × 2 Max	T	0.5	70.85 ± 11.00	64.51 ± 9.34	49.94 ± 8.82	86.72 ± 2.78	77.28 ± 5.71	84.08 ± 1.42
M5	2 × 2 Max	T	0.7	69.55 ± 9.81	62.55 ± 7.53	46.81 ± 6.19	81.16 ± 7.63	74.26 ± 2.78	83.59 ± 0.71
M5	2 × 2 Max	F	0.3	86.09 ± 1.91	77.55 ± 2.76	63.70 ± 4.33	76.21 ± 3.70	82.12 ± 2.65	86.73 ± 0.94
M5	2 × 2 Max	F	0.5	85.82 ± 0.20	76.29 ± 0.60	61.26 ± 1.30	70.31 ± 4.02	79.54 ± 1.63	86.14 ± 0.80
M5	2 × 2 Max	F	0.7	85.98 ± 2.04	78.02 ± 2.86	64.81 ± 4.65	80.68 ± 5.73	83.83 ± 3.40	86.84 ± 2.37
M5	2 × 2 Avg	T	0.3	60.07 ± 25.40	57.23 ± 17.82	44.26 ± 11.81	85.87 ± 16.33	70.52 ± 11.38	83.10 ± 3.31
M5	2 × 2 Avg	T	0.5	58.32 ± 12.09	54.56 ± 9.32	42.59 ± 5.64	94.20 ± 5.82	72.85 ± 5.55	86.72 ± 1.67
M5	2 × 2 Avg	T	0.7	53.32 ± 7.74	52.46 ± 6.32	41.28 ± 3.69	97.22 ± 2.44	72.29 ± 1.92	86.77 ± 1.48
M5	2 × 2 Avg	F	0.3	82.89 ± 1.79	74.58 ± 1.25	60.05 ± 1.88	80.31 ± 2.45	81.83 ± 1.27	88.73 ± 0.32
M5	2 × 2 Avg	F	0.5	86.09 ± 1.20	77.89 ± 0.76	64.42 ± 0.69	78.74 ± 4.46	83.12 ± 1.11	88.53 ± 0.51
M5	2 × 2 Avg	F	0.7	84.74 ± 3.08	76.73 ± 2.65	63.12 ± 3.08	80.92 ± 6.03	83.19 ± 0.88	89.08 ± 0.35
M5	3 × 3 Max	T	0.3	NA	NA	NA	NA	NA	NA
M5	3 × 3 Max	T	0.5	NA	NA	NA	NA	NA	NA
M5	3 × 3 Max	T	0.7	NA	NA	NA	NA	NA	NA
M5	3 × 3 Max	F	0.3	NA	NA	NA	NA	NA	NA
M5	3 × 3 Max	F	0.5	NA	NA	NA	NA	NA	NA
M5	3 × 3 Max	F	0.7	NA	NA	NA	NA	NA	NA
M5	3 × 3 Avg	T	0.3	NA	NA	NA	NA	NA	NA
M5	3 × 3 Avg	T	0.5	NA	NA	NA	NA	NA	NA
M5	3 × 3 Avg	T	0.7	NA	NA	NA	NA	NA	NA
M5	3 × 3 Avg	F	0.3	NA	NA	NA	NA	NA	NA
M5	3 × 3 Avg	F	0.5	NA	NA	NA	NA	NA	NA
M5	3 × 3 Avg	F	0.7	NA	NA	NA	NA	NA	NA

**Table A2:** Full-variable ablation results on the target-domain validation set using negative-class F1 as the selection metric. Values are mean ± std over three runs. Gray rows indicate representative configurations. NA denotes that the feature map collapsed to 0 × 0 with 3 × 3 pooling, causing a runtime error. T: Batch Normalization enabled; F: Batch Normalization disabled; DP: Dropout rate; Acc: Accuracy; MF1: Macro F1; NF1: Negative F1; NR: Negative Recall; MR: Macro Recall; AUC: Area Under the Curve.

Arch	Pooling	BN	DP	Acc (%)	MF1 (%)	NF1 (%)	NR (%)	MR (%)	AUC (%)
M1	2 × 2 Max	T	0.3	88.18 ± 1.18	79.15 ± 1.58	65.43 ± 2.47	70.06 ± 2.04	80.79 ± 1.18	88.24 ± 1.16
M1	2 × 2 Max	T	0.5	85.83 ± 1.97	56.74 ± 19.49	22.19 ± 38.43	26.09 ± 45.19	57.91 ± 19.72	64.77 ± 21.86
M1	2 × 2 Max	T	0.7	84.03 ± 0.00	45.66 ± 0.00	0.00 ± 0.00	0.00 ± 0.00	50.00 ± 0.00	49.89 ± 1.26
M1	2 × 2 Max	F	0.3	88.95 ± 0.72	78.78 ± 0.13	64.10 ± 0.36	61.84 ± 5.18	77.97 ± 1.65	88.40 ± 0.29
M1	2 × 2 Max	F	0.5	87.42 ± 1.08	77.31 ± 1.42	62.17 ± 2.65	64.99 ± 8.61	78.33 ± 3.22	87.92 ± 1.78
M1	2 × 2 Max	F	0.7	88.66 ± 1.54	78.70 ± 1.60	64.14 ± 2.31	63.29 ± 4.53	78.38 ± 1.37	88.62 ± 0.19
M1	2 × 2 Avg	T	0.3	87.44 ± 0.91	78.14 ± 1.14	63.88 ± 1.89	69.56 ± 5.00	80.21 ± 1.88	87.72 ± 1.66
M1	2 × 2 Avg	T	0.5	86.23 ± 1.07	76.18 ± 0.87	60.71 ± 1.47	66.79 ± 7.43	78.35 ± 2.55	87.73 ± 0.18
M1	2 × 2 Avg	T	0.7	84.03 ± 0.00	45.66 ± 0.00	0.00 ± 0.00	0.00 ± 0.00	50.00 ± 0.00	51.15 ± 1.35
M1	2 × 2 Avg	F	0.3	85.15 ± 1.15	75.72 ± 1.58	60.65 ± 3.01	71.85 ± 8.78	79.77 ± 3.36	88.95 ± 1.63
M1	2 × 2 Avg	F	0.5	87.69 ± 1.15	77.16 ± 1.59	61.67 ± 3.06	62.32 ± 9.44	77.42 ± 3.40	88.42 ± 0.44
M1	2 × 2 Avg	F	0.7	85.83 ± 1.71	74.57 ± 1.81	61.35 ± 0.14	65.40 ± 6.04	77.38 ± 0.85	87.54 ± 0.49
M1	3 × 3 Max	T	0.3	86.38 ± 1.65	74.00 ± 0.44	56.11 ± 1.29	54.83 ± 10.51	73.60 ± 3.17	85.96 ± 0.14
M1	3 × 3 Max	T	0.5	85.70 ± 1.96	73.94 ± 0.93	56.45 ± 0.98	57.97 ± 7.11	74.47 ± 0.18	87.42 ± 0.68
M1	3 × 3 Max	T	0.7	83.47 ± 3.11	72.97 ± 1.74	56.19 ± 1.50	66.06 ± 9.02	76.42 ± 2.33	86.21 ± 0.34
M1	3 × 3 Max	F	0.3	87.00 ± 1.22	75.81 ± 1.62	59.37 ± 2.91	59.66 ± 7.74	75.93 ± 2.86	84.88 ± 1.34
M1	3 × 3 Max	F	0.5	87.15 ± 2.26	77.29 ± 2.63	62.34 ± 3.68	66.31 ± 6.61	78.71 ± 2.43	87.63 ± 0.32
M1	3 × 3 Max	F	0.7	88.43 ± 1.24	77.60 ± 1.67	62.03 ± 2.63	59.06 ± 2.94	76.53 ± 1.25	87.31 ± 0.87
M1	3 × 3 Avg	T	0.3	86.42 ± 1.25	73.47 ± 1.36	54.93 ± 1.91	51.69 ± 0.75	72.36 ± 0.45	84.12 ± 1.92
M1	3 × 3 Avg	T	0.5	86.57 ± 1.61	74.13 ± 1.24	56.19 ± 1.38	53.71 ± 3.37	73.28 ± 0.46	84.74 ± 1.65
M1	3 × 3 Avg	T	0.7	84.22 ± 1.84	73.37 ± 1.10	56.40 ± 1.67	64.01 ± 8.11	76.03 ± 2.53	85.77 ± 0.56
M1	3 × 3 Avg	F	0.3	86.44 ± 2.02	74.34 ± 1.40	56.75 ± 2.73	56.04 ± 11.31	74.13 ± 3.76	84.48 ± 2.08
M1	3 × 3 Avg	F	0.5	84.70 ± 3.11	73.42 ± 1.91	56.17 ± 2.37	61.24 ± 11.03	75.20 ± 1.75	83.58 ± 0.99
M1	3 × 3 Avg	F	0.7	84.03 ± 0.00	45.66 ± 0.00	0.00 ± 0.00	0.00 ± 0.00	50.00 ± 0.00	41.17 ± 0.87
M2	2 × 2 Max	T	0.3	84.88 ± 1.58	74.60 ± 1.86	58.49 ± 2.63	66.42 ± 0.55	77.41 ± 1.13	84.64 ± 0.56
M2	2 × 2 Max	T	0.5	85.50 ± 1.52	75.27 ± 0.32	59.39 ± 0.53	66.55 ± 8.42	78.22 ± 2.52	85.48 ± 0.86
M2	2 × 2 Max	T	0.7	86.03 ± 1.61	75.24 ± 1.85	58.92 ± 3.59	62.92 ± 8.97	76.67 ± 3.48	85.30 ± 1.50
M2	2 × 2 Max	F	0.3	89.27 ± 0.37	80.52 ± 0.54	67.46 ± 1.31	69.81 ± 3.21	81.39 ± 2.36	87.03 ± 0.84
M2	2 × 2 Max	F	0.5	88.43 ± 0.99	79.96 ± 0.62	66.94 ± 0.78	73.31 ± 5.10	82.27 ± 1.56	87.17 ± 0.31
M2	2 × 2 Max	F	0.7	88.91 ± 1.17	80.38 ± 1.07	67.46 ± 1.97	72.22 ± 9.67	82.15 ± 3.49	87.09 ± 0.31
M2	2 × 2 Avg	T	0.3	87.44 ± 2.55	78.43 ± 1.89	64.51 ± 2.67	71.40 ± 12.39	80.94 ± 3.99	87.66 ± 0.81
M2	2 × 2 Avg	T	0.5	87.08 ± 1.08	78.09 ± 1.03	64.06 ± 1.34	72.00 ± 2.59	80.99 ± 0.63	86.84 ± 0.34
M2	2 × 2 Avg	T	0.7	86.27 ± 2.02	76.48 ± 1.80	61.34 ± 3.27	68.35 ± 10.63	79.01 ± 3.84	87.10 ± 1.00
M2	2 × 2 Avg	F	0.3	87.71 ± 1.27	79.18 ± 1.49	65.86 ± 2.15	73.93 ± 2.12	82.22 ± 0.85	87.67 ± 0.81
M2	2 × 2 Avg	F	0.5	87.81 ± 1.00	78.63 ± 0.63	64.62 ± 0.73	69.69 ± 5.86	80.47 ± 1.55	87.50 ± 0.66
M2	2 × 2 Avg	F	0.7	88.52 ± 0.35	78.63 ± 0.62	64.09 ± 1.03	64.13 ± 0.87	78.63 ± 0.62	86.40 ± 0.48
M2	3 × 3 Max	T	0.3	85.30 ± 1.55	73.02 ± 1.01	54.83 ± 1.22	55.80 ± 4.95	73.35 ± 1.24	83.19 ± 1.11
M2	3 × 3 Max	T	0.5	85.22 ± 1.57	73.01 ± 2.71	54.86 ± 4.66	56.28 ± 6.23	73.50 ± 3.00	83.87 ± 3.60
M2	3 × 3 Max	T	0.7	85.28 ± 2.12	73.03 ± 2.94	54.86 ± 4.49	55.67 ± 1.93	73.30 ± 2.23	83.97 ± 3.04
M2	3 × 3 Max	F	0.3	86.25 ± 0.79	75.07 ± 1.50	58.32 ± 2.49	60.26 ± 1.66	75.72 ± 1.66	86.88 ± 0.63
M2	3 × 3 Max	F	0.5	87.50 ± 0.44	77.25 ± 0.61	62.00 ± 1.44	64.01 ± 6.34	77.97 ± 2.32	88.64 ± 0.29
M2	3 × 3 Max	F	0.7	85.74 ± 0.09	75.15 ± 0.38	58.92 ± 0.76	64.01 ± 1.57	76.95 ± 0.74	87.72 ± 0.45
M2	3 × 3 Avg	T	0.3	85.30 ± 1.45	73.19 ± 1.12	55.18 ± 1.27	56.52 ± 2.73	73.61 ± 0.29	84.53 ± 0.99
M2	3 × 3 Avg	T	0.5	85.78 ± 1.71	73.50 ± 1.76	55.47 ± 2.35	55.20 ± 1.60	73.40 ± 0.54	84.21 ± 1.40
M2	3 × 3 Avg	T	0.7	85.23 ± 0.69	73.56 ± 1.25	56.00 ± 2.22	58.90 ± 3.55	74.58 ± 1.69	84.13 ± 2.16
M2	3 × 3 Avg	F	0.3	84.03 ± 0.00	45.66 ± 0.00	0.00 ± 0.00	0.00 ± 0.00	50.00 ± 0.00	40.42 ± 0.55
M2	3 × 3 Avg	F	0.5	84.03 ± 0.00	45.66 ± 0.00	0.00 ± 0.00	0.00 ± 0.00	50.00 ± 0.00	40.45 ± 0.83
M2	3 × 3 Avg	F	0.7	84.03 ± 0.00	45.66 ± 0.00	0.00 ± 0.00	0.00 ± 0.00	50.00 ± 0.00	40.53 ± 0.63
M3	2 × 2 Max	T	0.3	86.32 ± 0.97	75.92 ± 1.47	60.11 ± 3.22	65.10 ± 11.25	77.73 ± 4.18	87.37 ± 1.81
M3	2 × 2 Max	T	0.5	81.12 ± 1.03	72.56 ± 0.31	57.26 ± 1.41	79.47 ± 8.67	80.45 ± 2.88	87.26 ± 1.35
M3	2 × 2 Max	T	0.7	83.83 ± 2.93	74.83 ± 1.69	59.82 ± 1.10	75.03 ± 10.04	80.26 ± 2.03	87.22 ± 0.62
M3	2 × 2 Max	F	0.3	89.04 ± 1.19	81.05 ± 0.84	68.78 ± 0.90	75.36 ± 5.34	83.50 ± 1.43	88.56 ± 1.14
M3	2 × 2 Max	F	0.5	88.56 ± 0.91	80.81 ± 1.48	68.62 ± 2.55	78.39 ± 5.62	84.44 ± 2.45	88.83 ± 0.53
M3	2 × 2 Max	F	0.7	90.90 ± 0.64	82.91 ± 1.62	71.23 ± 2.97	70.89 ± 6.69	82.79 ± 2.97	88.39 ± 0.41
M3	2 × 2 Avg	T	0.3	81.69 ± 0.96	72.72 ± 0.76	57.07 ± 1.13	76.21 ± 4.28	79.47 ± 1.38	87.07 ± 0.50
M3	2 × 2 Avg	T	0.5	77.16 ± 2.97	68.27 ± 1.62	51.56 ± 2.48	76.45 ± 12.44	76.88 ± 3.98	85.80 ± 2.57
M3	2 × 2 Avg	T	0.7	83.41 ± 1.05	74.01 ± 1.68	58.39 ± 2.80	72.95 ± 5.04	79.18 ± 2.44	85.43 ± 0.84
M3	2 × 2 Avg	F	0.3	87.27 ± 1.08	77.97 ± 2.17	63.66 ± 3.96	70.31 ± 10.63	80.40 ± 3.32	86.02 ± 1.85
M3	2 × 2 Avg	F	0.5	86.61 ± 2.62	76.96 ± 1.94	62.07 ± 2.33	68.16 ± 8.17	79.12 ± 2.03	85.69 ± 1.02
M3	2 × 2 Avg	F	0.7	87.15 ± 0.79	78.15 ± 1.62	64.13 ± 2.86	72.10 ± 5.36	81.06 ± 2.51	86.92 ± 0.53
M3	3 × 3 Max	T	0.3	NA	NA	NA	NA	NA	NA
M3	3 × 3 Max	T	0.5	NA	NA	NA	NA	NA	NA
M3	3 × 3 Max	T	0.7	NA	NA	NA	NA	NA	NA
M3	3 × 3 Max	F	0.3	NA	NA	NA	NA	NA	NA
M3	3 × 3 Max	F	0.5	NA	NA	NA	NA	NA	NA
M3	3 × 3 Max	F	0.7	NA	NA	NA	NA	NA	NA
M3	3 × 3 Avg	T	0.3	NA	NA	NA	NA	NA	NA
M3	3 × 3 Avg	T	0.5	NA	NA	NA	NA	NA	NA
M3	3 × 3 Avg	T	0.7	NA	NA	NA	NA	NA	NA
M3	3 × 3 Avg	F	0.3	NA	NA	NA	NA	NA	NA
M3	3 × 3 Avg	F	0.5	NA	NA	NA	NA	NA	NA
M3	3 × 3 Avg	F	0.7	NA	NA	NA	NA	NA	NA
M4	2 × 2 Max	T	0.3	84.18 ± 2.55	73.61 ± 2.91	56.92 ± 4.35	64.98 ± 2.72	76.40 ± 1.88	85.78 ± 1.28
M4	2 × 2 Max	T	0.5	82.70 ± 2.56	71.71 ± 0.47	54.16 ± 2.00	64.61 ± 13.89	75.37 ± 4.12	85.95 ± 1.73
M4	2 × 2 Max	T	0.7	84.88 ± 1.73	73.41 ± 0.85	56.00 ± 2.46	60.75 ± 11.67	75.11 ± 3.76	85.80 ± 0.46
M4	2 × 2 Max	F	0.3	90.16 ± 1.12	81.29 ± 1.43	68.41 ± 2.19	66.55 ± 2.96	80.60 ± 1.01	87.33 ± 0.20
M4	2 × 2 Max	F	0.5	88.79 ± 1.65	80.46 ± 1.78	67.72 ± 2.44	73.19 ± 3.14	82.47 ± 0.59	88.59 ± 1.23
M4	2 × 2 Max	F	0.7	89.39 ± 1.23	80.66 ± 2.03	67.68 ± 3.37	69.45 ± 2.79	81.31 ± 1.84	87.31 ± 0.76
M4	2 × 2 Avg	T	0.3	86.00 ± 2.14	76.45 ± 1.93	61.48 ± 2.77	69.81 ± 7.68	79.44 ± 2.41	88.32 ± 1.33
M4	2 × 2 Avg	T	0.5	85.69 ± 2.83	74.75 ± 1.01	58.19 ± 1.26	62.56 ± 13.23	76.32 ± 3.79	87.48 ± 1.11
M4	2 × 2 Avg	T	0.7	84.99 ± 1.79	74.39 ± 0.22	57.94 ± 1.00	61.64 ± 8.95	76.78 ± 3.05	87.10 ± 0.57
M4	2 × 2 Avg	F	0.3	85.60 ± 2.53	76.19 ± 1.84	61.26 ± 1.27	70.77 ± 7.72	79.60 ± 1.74	88.53 ± 1.43
M4	2 × 2 Avg	F	0.5	86.55 ± 1.42	76.87 ± 1.33	61.93 ± 3.11	69.20 ± 13.23	79.53 ± 4.82	87.25 ± 2.17
M4	2 × 2 Avg	F	0.7	86.40 ± 3.24	76.66 ± 1.73	61.66 ± 1.70	68.12 ± 12.84	79.00 ± 3.48	88.63 ± 1.17
M4	3 × 3 Max	T	0.3	NA	NA	NA	NA	NA	NA
M4	3 × 3 Max	T	0.5	NA	NA	NA	NA	NA	NA
M4	3 × 3 Max	T	0.7	NA	NA	NA	NA	NA	NA
M4	3 × 3 Max	F	0.3	NA	NA	NA	NA	NA	NA
M4	3 × 3 Max	F	0.5	NA	NA	NA	NA	NA	NA
M4	3 × 3 Max	F	0.7	NA	NA	NA	NA	NA	NA
M4	3 × 3 Avg	T	0.3	NA	NA	NA	NA	NA	NA
M4	3 × 3 Avg	T	0.5	NA	NA	NA	NA	NA	NA
M4	3 × 3 Avg	T	0.7	NA	NA	NA	NA	NA	NA
M4	3 × 3 Avg	F	0.3	NA	NA	NA	NA	NA	NA
M4	3 × 3 Avg	F	0.5	NA	NA	NA	NA	NA	NA
M4	3 × 3 Avg	F	0.7	NA	NA	NA	NA	NA	NA
M5	2 × 2 Max	T	0.3	83.41 ± 3.63	72.70 ± 3.10	55.70 ± 5.25	65.94 ± 16.38	76.34 ± 4.83	85.33 ± 2.34
M5	2 × 2 Max	T	0.5	80.15 ± 4.17	71.05 ± 2.72	54.93 ± 3.21	75.61 ± 14.47	78.31 ± 4.21	84.13 ± 1.40
M5	2 × 2 Max	T	0.7	84.61 ± 0.26	72.24 ± 0.73	53.72 ± 1.59	56.04 ± 4.04	73.04 ± 1.51	84.06 ± 1.08
M5	2 × 2 Max	F	0.3	89.31 ± 1.13	80.86 ± 0.29	68.17 ± 0.99	71.86 ± 9.88	82.25 ± 3.57	87.90 ± 0.39
M5	2 × 2 Max	F	0.5	89.41 ± 1.65	79.33 ± 1.25	64.91 ± 1.53	61.11 ± 6.76	77.94 ± 2.13	87.18 ± 0.53
M5	2 × 2 Max	F	0.7	88.16 ± 1.13	80.25 ± 0.78	67.78 ± 1.13	78.02 ± 7.75	84.05 ± 3.89	88.36 ± 0.37
M5	2 × 2 Avg	T	0.3	84.55 ± 1.48	72.62 ± 1.23	54.57 ± 2.51	58.33 ± 8.43	73.93 ± 1.82	84.67 ± 0.67
M5	2 × 2 Avg	T	0.5	82.27 ± 3.06	72.63 ± 1.54	56.46 ± 0.82	71.74 ± 10.81	78.01 ± 2.36	85.92 ± 1.80
M5	2 × 2 Avg	T	0.7	79.59 ± 3.64	71.34 ± 3.17	55.99 ± 3.64	80.56 ± 3.27	79.97 ± 1.48	86.50 ± 2.41
M5	2 × 2 Avg	F	0.3	86.42 ± 3.90	78.08 ± 4.27	64.58 ± 5.94	75.81 ± 3.29	82.14 ± 1.23	88.77 ± 0.39
M5	2 × 2 Avg	F	0.5	87.25 ± 0.57	78.74 ± 0.65	65.30 ± 0.78	75.12 ± 3.92	82.34 ± 1.33	88.39 ± 1.08
M5	2 × 2 Avg	F	0.7	87.81 ± 1.83	79.10 ± 2.78	65.60 ± 4.38	72.58 ± 3.82	81.64 ± 2.17	89.11 ± 0.31
M5	3 × 3 Max	T	0.3	NA	NA	NA	NA	NA	NA
M5	3 × 3 Max	T	0.5	NA	NA	NA	NA	NA	NA
M5	3 × 3 Max	T	0.7	NA	NA	NA	NA	NA	NA
M5	3 × 3 Max	F	0.3	NA	NA	NA	NA	NA	NA
M5	3 × 3 Max	F	0.5	NA	NA	NA	NA	NA	NA
M5	3 × 3 Max	F	0.7	NA	NA	NA	NA	NA	NA
M5	3 × 3 Avg	T	0.3	NA	NA	NA	NA	NA	NA
M5	3 × 3 Avg	T	0.5	NA	NA	NA	NA	NA	NA
M5	3 × 3 Avg	T	0.7	NA	NA	NA	NA	NA	NA
M5	3 × 3 Avg	F	0.3	NA	NA	NA	NA	NA	NA
M5	3 × 3 Avg	F	0.5	NA	NA	NA	NA	NA	NA
M5	3 × 3 Avg	F	0.7	NA	NA	NA	NA	NA	NA

**Table A3:** Class imbalance and post-processing ablation on the target-domain validation set. Values are mean ± std over three runs. CW: class weight; γ: focal gamma; TT: threshold tuning. Acc: Accuracy; MF1: Macro F1; NF1: Negative F1; NR: Negative Recall; MR: Macro Recall; AUC: Area Under the Curve.

Arch	CW	γ	TT	Acc (%)	MF1 (%)	NF1 (%)	NR (%)	MR (%)	AUC (%)
M3_dp0.7	1	0	F	89.35 ± 1.09	81.37 ± 1.24	69.18 ± 1.99	74.88 ± 6.81	83.49 ± 2.51	88.82 ± 0.65
M3_dp0.7	1	0	T	89.93 ± 0.72	81.83 ± 1.20	69.69 ± 2.07	72.59 ± 5.09	82.91 ± 2.14	88.82 ± 0.65
M3_dp0.7	1	2	F	89.60 ± 1.37	81.44 ± 1.62	69.13 ± 2.30	72.59 ± 1.82	82.71 ± 0.09	87.99 ± 0.74
M3_dp0.7	1	2	T	89.89 ± 1.28	81.99 ± 1.99	70.06 ± 3.12	73.91 ± 2.37	83.42 ± 1.56	87.99 ± 0.74
M3_dp0.7	3	0	F	88.08 ± 0.68	79.23 ± 0.84	65.68 ± 1.26	71.38 ± 1.92	81.32 ± 0.79	87.09 ± 0.53
M3_dp0.7	3	0	T	89.06 ± 2.13	80.66 ± 2.04	67.91 ± 2.71	71.86 ± 5.85	82.10 ± 1.28	87.09 ± 0.53
M3_dp0.7	3	2	F	87.73 ± 1.27	78.78 ± 1.10	64.97 ± 1.20	71.14 ± 3.63	81.01 ± 0.78	86.13 ± 1.74
M3_dp0.7	3	2	T	88.73 ± 0.63	79.71 ± 0.68	66.18 ± 0.94	68.97 ± 1.85	80.73 ± 0.33	86.13 ± 1.74
M3_dp0.7	5	0	F	87.44 ± 0.67	78.68 ± 1.89	64.95 ± 3.50	73.19 ± 7.30	81.67 ± 3.34	86.37 ± 0.57
M3_dp0.7	5	0	T	88.12 ± 0.59	80.14 ± 0.54	67.55 ± 0.57	77.42 ± 2.30	83.78 ± 0.62	86.37 ± 0.57
M3_dp0.7	5	2	F	87.48 ± 0.51	78.84 ± 0.42	65.32 ± 0.52	73.79 ± 2.01	81.94 ± 0.52	86.42 ± 1.23
M3_dp0.7	5	2	T	88.16 ± 0.64	79.33 ± 0.63	65.83 ± 0.73	71.38 ± 1.66	81.36 ± 0.42	86.42 ± 1.23
M3_dp0.3	1	0	F	89.04 ± 1.19	81.05 ± 0.84	68.78 ± 0.90	75.36 ± 5.34	83.50 ± 1.43	88.56 ± 1.14
M3_dp0.3	1	0	T	90.03 ± 0.35	82.06 ± 0.43	70.10 ± 0.66	73.19 ± 1.35	83.21 ± 0.45	88.56 ± 1.14
M3_dp0.3	1	2	F	88.56 ± 0.61	79.86 ± 0.70	66.64 ± 1.94	71.98 ± 9.90	81.85 ± 3.66	84.43 ± 5.46
M3_dp0.3	1	2	T	88.93 ± 0.33	80.21 ± 1.12	67.10 ± 2.45	71.14 ± 9.82	81.72 ± 3.50	84.43 ± 5.46
M3_dp0.3	3	0	F	87.81 ± 0.54	78.95 ± 0.38	65.30 ± 0.66	71.86 ± 3.86	81.35 ± 1.30	86.05 ± 0.46
M3_dp0.3	3	0	T	88.47 ± 0.17	79.85 ± 0.33	66.66 ± 0.58	72.22 ± 1.07	81.89 ± 0.81	86.05 ± 0.46
M3_dp0.3	3	2	F	87.52 ± 1.56	78.43 ± 0.97	64.44 ± 0.85	70.65 ± 6.44	80.69 ± 1.46	86.83 ± 1.47
M3_dp0.3	3	2	T	89.16 ± 1.26	79.96 ± 1.35	66.39 ± 1.93	66.79 ± 2.57	80.10 ± 0.33	86.83 ± 1.47
M3_dp0.3	5	0	F	87.50 ± 0.97	79.48 ± 1.02	66.66 ± 1.23	78.14 ± 3.00	83.71 ± 0.90	86.48 ± 1.04
M3_dp0.3	5	0	T	87.89 ± 0.60	80.00 ± 0.28	67.45 ± 0.44	78.62 ± 4.37	84.13 ± 1.49	86.48 ± 1.04
M3_dp0.3	5	2	F	88.25 ± 1.03	79.71 ± 1.33	66.55 ± 2.02	73.07 ± 3.29	82.10 ± 1.24	87.25 ± 1.43
M3_dp0.3	5	2	T	88.66 ± 1.48	80.19 ± 1.60	67.25 ± 2.24	72.59 ± 2.46	82.15 ± 0.30	87.25 ± 1.43
M3_dp0.5	1	0	F	89.52 ± 1.10	82.07 ± 0.70	70.44 ± 0.66	78.02 ± 6.03	84.87 ± 1.60	88.65 ± 0.83
M3_dp0.5	1	0	T	90.45 ± 1.42	82.58 ± 0.68	70.89 ± 0.46	72.71 ± 9.83	83.27 ± 3.14	88.65 ± 0.83
M3_dp0.5	1	2	F	90.12 ± 1.93	82.12 ± 1.93	70.16 ± 2.60	72.10 ± 5.98	82.83 ± 1.40	87.88 ± 0.57
M3_dp0.5	1	2	T	90.74 ± 1.29	82.86 ± 1.66	71.24 ± 2.32	71.50 ± 3.04	83.03 ± 0.58	87.88 ± 0.57
M3_dp0.5	3	0	F	86.44 ± 1.30	77.65 ± 1.14	63.65 ± 1.55	74.28 ± 5.53	81.51 ± 1.83	86.72 ± 1.95
M3_dp0.5	3	0	T	87.88 ± 1.30	79.00 ± 1.66	65.32 ± 2.46	71.26 ± 1.04	81.15 ± 0.89	86.72 ± 1.95
M3_dp0.5	3	2	F	88.56 ± 0.98	79.80 ± 1.55	66.50 ± 2.72	71.26 ± 6.49	81.55 ± 2.72	86.85 ± 0.98
M3_dp0.5	3	2	T	88.27 ± 1.23	79.86 ± 1.48	66.85 ± 2.45	73.96 ± 4.58	82.51 ± 2.19	86.85 ± 0.98
M3_dp0.5	5	0	F	87.15 ± 2.06	78.76 ± 2.00	65.42 ± 2.59	75.60 ± 3.81	82.48 ± 0.57	86.14 ± 0.92
M3_dp0.5	5	0	T	87.69 ± 1.34	79.39 ± 1.49	66.31 ± 2.13	75.60 ± 1.50	82.80 ± 0.53	86.14 ± 0.92
M3_dp0.5	5	2	F	86.92 ± 2.10	79.26 ± 1.77	66.69 ± 2.00	81.52 ± 7.24	84.77 ± 1.74	88.40 ± 0.89
M3_dp0.5	5	2	T	88.62 ± 1.68	80.99 ± 1.20	68.97 ± 1.03	78.86 ± 7.73	84.67 ± 2.09	88.40 ± 0.89
M4_dp0.3	1	0	F	89.31 ± 2.06	80.59 ± 2.28	67.58 ± 3.18	69.10 ± 3.49	81.12 ± 0.31	87.62 ± 0.84
M4_dp0.3	1	0	T	89.06 ± 2.27	80.76 ± 2.36	68.15 ± 3.21	72.47 ± 4.86	82.34 ± 0.76	87.62 ± 0.84
M4_dp0.3	1	2	F	89.58 ± 2.05	81.11 ± 2.36	68.46 ± 3.44	70.17 ± 2.28	81.72 ± 0.36	87.66 ± 0.59
M4_dp0.3	1	2	T	89.43 ± 1.29	81.24 ± 2.01	68.85 ± 3.20	73.07 ± 5.36	82.80 ± 2.26	87.66 ± 0.59
M4_dp0.3	3	0	F	88.52 ± 1.08	79.65 ± 1.02	66.22 ± 1.27	70.29 ± 2.46	81.14 ± 0.40	87.55 ± 1.08
M4_dp0.3	3	0	T	88.37 ± 1.35	79.64 ± 1.03	66.31 ± 1.13	71.50 ± 4.39	81.54 ± 1.08	87.55 ± 1.08
M4_dp0.3	3	2	F	89.14 ± 0.49	79.67 ± 0.58	65.79 ± 0.97	65.46 ± 4.29	79.55 ± 1.57	87.64 ± 0.76
M4_dp0.3	3	2	T	89.31 ± 0.49	80.44 ± 0.76	67.26 ± 1.26	68.72 ± 2.20	80.97 ± 0.95	87.64 ± 0.76
M4_dp0.3	5	0	F	88.75 ± 0.79	79.36 ± 1.12	65.43 ± 1.69	66.55 ± 0.55	79.76 ± 0.66	85.10 ± 0.58
M4_dp0.3	5	0	T	89.10 ± 0.57	79.81 ± 1.10	66.10 ± 1.85	66.55 ± 2.64	80.00 ± 1.27	85.10 ± 0.58
M4_dp0.3	5	2	F	86.74 ± 2.89	78.03 ± 3.07	64.22 ± 4.22	73.67 ± 6.27	81.45 ± 1.89	85.76 ± 2.02
M4_dp0.3	5	2	T	87.81 ± 2.85	79.06 ± 3.03	65.54 ± 4.79	71.62 ± 5.85	81.26 ± 1.54	85.76 ± 2.02
M5_dp0.3	1	0	F	89.31 ± 1.13	80.86 ± 0.29	68.17 ± 0.99	71.86 ± 9.88	82.25 ± 3.57	87.90 ± 0.39
M5_dp0.3	1	0	T	90.22 ± 1.05	81.79 ± 0.53	69.42 ± 1.15	69.69 ± 9.31	81.90 ± 3.57	87.90 ± 0.39
M5_dp0.3	1	2	F	88.33 ± 1.06	79.60 ± 0.99	66.27 ± 1.44	71.74 ± 5.23	81.61 ± 1.66	86.74 ± 0.39
M5_dp0.3	1	2	T	88.91 ± 0.89	80.20 ± 0.87	67.08 ± 1.38	70.65 ± 5.18	81.52 ± 0.74	86.74 ± 0.39
M5_dp0.3	3	0	F	89.76 ± 1.36	81.73 ± 1.56	69.62 ± 2.31	73.19 ± 2.37	83.06 ± 0.67	88.18 ± 0.54
M5_dp0.3	3	0	T	89.91 ± 1.11	82.05 ± 1.50	70.18 ± 2.38	74.28 ± 5.45	83.58 ± 2.18	88.18 ± 0.54
M5_dp0.3	3	2	F	88.03 ± 1.22	79.57 ± 0.67	66.43 ± 0.42	74.16 ± 6.54	82.41 ± 2.13	87.24 ± 0.59
M5_dp0.3	3	2	T	88.95 ± 0.96	80.45 ± 0.68	67.58 ± 0.94	72.10 ± 5.74	82.13 ± 1.97	87.24 ± 0.59
M5_dp0.3	5	0	F	86.82 ± 1.37	77.26 ± 2.69	62.51 ± 4.54	68.97 ± 6.55	79.59 ± 3.48	85.00 ± 3.66
M5_dp0.3	5	0	T	88.48 ± 1.32	78.80 ± 2.87	64.47 ± 4.99	65.70 ± 7.08	79.25 ± 3.61	85.00 ± 3.66
M5_dp0.3	5	2	F	85.78 ± 1.62	76.78 ± 1.31	62.33 ± 1.46	73.43 ± 3.98	80.78 ± 0.73	86.38 ± 0.35
M5_dp0.3	5	2	T	86.61 ± 2.07	77.58 ± 1.81	63.37 ± 2.23	72.10 ± 4.55	80.74 ± 0.47	86.38 ± 0.35
